# Warm, dry winters truncate timing and size distribution of seaward‐migrating salmon across a large, regulated watershed

**DOI:** 10.1002/eap.1880

**Published:** 2019-04-08

**Authors:** Stuart H. Munsch, Correigh M. Greene, Rachel C. Johnson, William H. Satterthwaite, Hiroo Imaki, Patricia L. Brandes

**Affiliations:** ^1^ Ocean Associates Inc. Under Contract to Northwest Fisheries Science Center National Marine Fisheries Service, NOAA 2725 Montlake Boulevard East Seattle Washington 98112 USA; ^2^ Fish Ecology Division Northwest Fisheries Science Center National Marine Fisheries Service, NOAA 2725 Montlake Boulevard East Seattle Washington 98112 USA; ^3^ Fisheries Ecology Division Southwest Fisheries Science Center National Marine Fisheries Service, NOAA 110 McAllister Way Santa Cruz California 95060 USA; ^4^ Center for Watershed Sciences University of California Davis 1 Shields Avenue Davis California 95616 USA; ^5^ U.S. Fish and Wildlife Service 850 S. Guild Avenue, Suite 105 Lodi California 95240 USA

**Keywords:** dams, drought, flow, migration, nursery, phenology, reservoirs, salmonids, snow, temperature mitigation, thermal tolerance

## Abstract

Ecologists are pressed to understand how climate constrains the timings of annual biological events (phenology). Climate influences on phenology are likely significant in estuarine watersheds because many watersheds provide seasonal fish nurseries where juvenile presence is synched with favorable conditions. While ecologists have long recognized that estuaries are generally important to juvenile fish, we incompletely understand the specific ecosystem dynamics that contribute to their nursery habitat value, limiting our ability to identify and protect vital habitat components. Here we examined the annual timing of juvenile coldwater fish migrating through a seasonally warm, hydrologically managed watershed. Our goal was to (1) understand how climate constrained the seasonal timing of water conditions necessary for juvenile fish to use nursery habitats and (2) inform management decisions about (a) mitigating climate‐mediated stress on nursery habitat function and (b) conserving heat‐constrained species in warming environments. Cool, wet winters deposited snow and cold water into mountains and reservoirs, which kept the lower watershed adequately cool for juveniles through the spring despite the region approaching its hot, dry summers. For every 1°C waters in April were colder, the juvenile fish population (1) inhabited the watershed 4–7 d longer and (2) entered marine waters, where survival is size selective, at maximum sizes 2.1 mm larger. Climate therefore appeared to constrain the nursery functions of this system by determining seasonal windows of tolerable rearing conditions, and cold water appeared to be a vital ecosystem component that promoted juvenile rearing. Fish in this system inhabit the southernmost extent of their range and already rear during the coolest part of the year, suggesting that a warming climate will truncate rather than shift their annual presence. Our findings are concerning for coldwater diadromous species in general because warming climates may constrain watershed use and diminish viability of life histories (e.g., late springtime rearing) and associated portfolio benefits over the long term. Lower watershed nurseries for coldwater fish in warming climates may be enhanced through allocating coldwater reservoir releases to prolong juvenile rearing periods downstream or restorations that facilitate colder conditions.

## Introduction

Many taxa migrate to track favorable conditions that vary in time and space. Reproduction is often timed in migratory life histories so that juveniles can exploit conditions that promote growth and survival (e.g., Van Der Jeugd et al. 2009). Anadromy is an example of this strategy, whereby juveniles can rear initially in watersheds and grow before migrating to sea where growth potential is higher, but predation risk is also high and dependent on size (Quinn 2005). Lower watershed components such as estuaries are often important habitats for migratory fish because they offer high densities of small prey to fuel growth and migration (Kjelson et al. 1982, Beck et al. 2001). The conditions of riverine and estuarine watershed components vary among seasons and some anadromous life histories exploit springtime conditions of watersheds to rear when, typically, prey availability is high, predation risks are comparatively low, habitats are inundated and flowing, and temperatures facilitate metabolism conducive to growth (Quinn 2005). This allows fish to emigrate over the spring and summer to marine environments, where prey are also seasonally abundant, and rapid early growth promotes marine survival (Woodson et al. 2013). Thus, anadromy benefits fish by synchronizing juvenile phases with optimal seasonal conditions.

The condition of regional environments can influence migration timing. For example, warmer springs can advance the arrival of migratory birds on nesting grounds (Bradley et al. 1999), and early wet seasons and high soil moisture during dry seasons can advance migrations of butterflies (Srygley et al. 2010). Similar dynamics occur for anadromous species. Warmer summer temperatures can advance summer migrations of anadromous adults into fresh waters (Quinn and Adams 1996) and high river flows can force or induce juvenile migrations downstream en route to the ocean. (Kjelson et al. 1982). Such phenologies are of conservation interest because the timings of many ecological events are responding to long‐term changes in environmental conditions (e.g., Bradley et al. 1999).

Anthropogenic changes in the timing of natural processes have substantial potential to alter migration timing. In many watersheds, snowpack is a natural reservoir that disperses cool, snow‐fed runoff throughout the landscape in the spring and summer (e.g., Knowles and Cayan 2002). In addition, dams and artificial reservoirs have proliferated globally and, by retaining waters, altered the timing and magnitude of downstream flow and temperature (Olden and Naiman 2009, Couto and Olden 2018). In some watersheds, managers can control the amount and temperature (by sourcing water from portions of thermoclines) of waters released from reservoirs to facilitate favorable conditions for fish downstream (e.g., Danner et al. 2012). The water temperatures stored by reservoirs and thus available for release depend on factors such as recent air temperature and precipitation (Nickel et al. 2004). However, in many regions, air temperatures are rising (Knowles and Cayan 2002, Barnett et al. 2005), springtime snowpacks are decreasing (Mote et al. 2018), and lake and reservoir temperatures are rising (O'Reilly et al. 2015). Thus, the timing and persistence of water conditions favorable for cold‐water migratory species are potentially governed by changing climates and hydrologic modifications.

Climate may mediate the nursery value of watersheds by constraining migration timing of juvenile fish. Ecologists are recognizing that the value of nursery habitats should be measured by their ability to support population dynamics including ontogenetic migrations that allow fish to access appropriate environments given their developmental stage (Sheaves et al. 2015). Coldwater anadromous fish rear inland within a diversity of climates, including areas that approach thermal limits (Kjelson et al. 1982, Quinn 2005, Richter and Kolmes 2005), and climate‐driven variation in watershed conditions (e.g., flow, temperature) among years can determine the survival of juvenile anadromous fish (Crozier and Zabel 2006). It remains less clear, however, how climate may constrain the timing of ontogenetic migrations by determining annual windows within which juveniles can access rearing habitats. This issue is especially relevant to coldwater fish in warmer regions, which may not be able to shift their timing in response to changes in climate conditions, but rather compress their timing during critical juvenile stages (sensu Mantua et al. 2015). The extent of seasonal time windows that support appropriate habitat conditions is significant because the anadromous life history template (i.e., migration between fresh and marine waters) includes variants characterized by differences in their timing and residencies among habitat types. For example, some life histories rear in watersheds late in the spring, provided that the watershed remains inhabitable. A diversity of life history variants is beneficial because it disperses fish and integrates stochastic habitat experiences across time and space, minimizing competition (Greene et al. 2010) and spreading risk (Schindler et al. 2010). By understanding how climate, hydrology, and managed water infrastructure determine when juvenile fish can exploit rearing habitats, we can better appreciate how these factors influence nursery habitat value in individual years and constrain the viability of life history diversity over many years.

Here we quantified relationships among regional winter weather, springtime snowpack and reservoir conditions, springtime stream temperature and flow, and annual outmigration timing and maximum sizes of juvenile anadromous fish in a lower watershed. These fish begin using the watershed in the winter but are sensitive to warm waters that occurred as precipitation declined and temperatures rose regionally in the summer. We hypothesized that cold, wet winters would store an abundance of snowpack in the mountains and cold water in reservoirs, which would prolong the presence of cold, high‐flowing waters downstream in the spring. We further hypothesized that these cool, flowing conditions persisting into the spring would allow juvenile fish to inhabit the watershed later in the year and emigrate to sea at larger sizes. We can expect air temperatures to rise and snowpack to decline in many systems (e.g., Barnett et al. 2005) and conservation of fish nurseries must be improved by understanding when and why juveniles use nursery habitats (Sheaves et al. 2015). Accordingly, our goal was to use field‐based observations to understand how regional climates, hydrologic infrastructure, and physiological limits of fish can determine the timing of limiting habitat conditions and, by implication, the nursery functions of these habitats.

## Methods

### Study system

**Figure 1:**
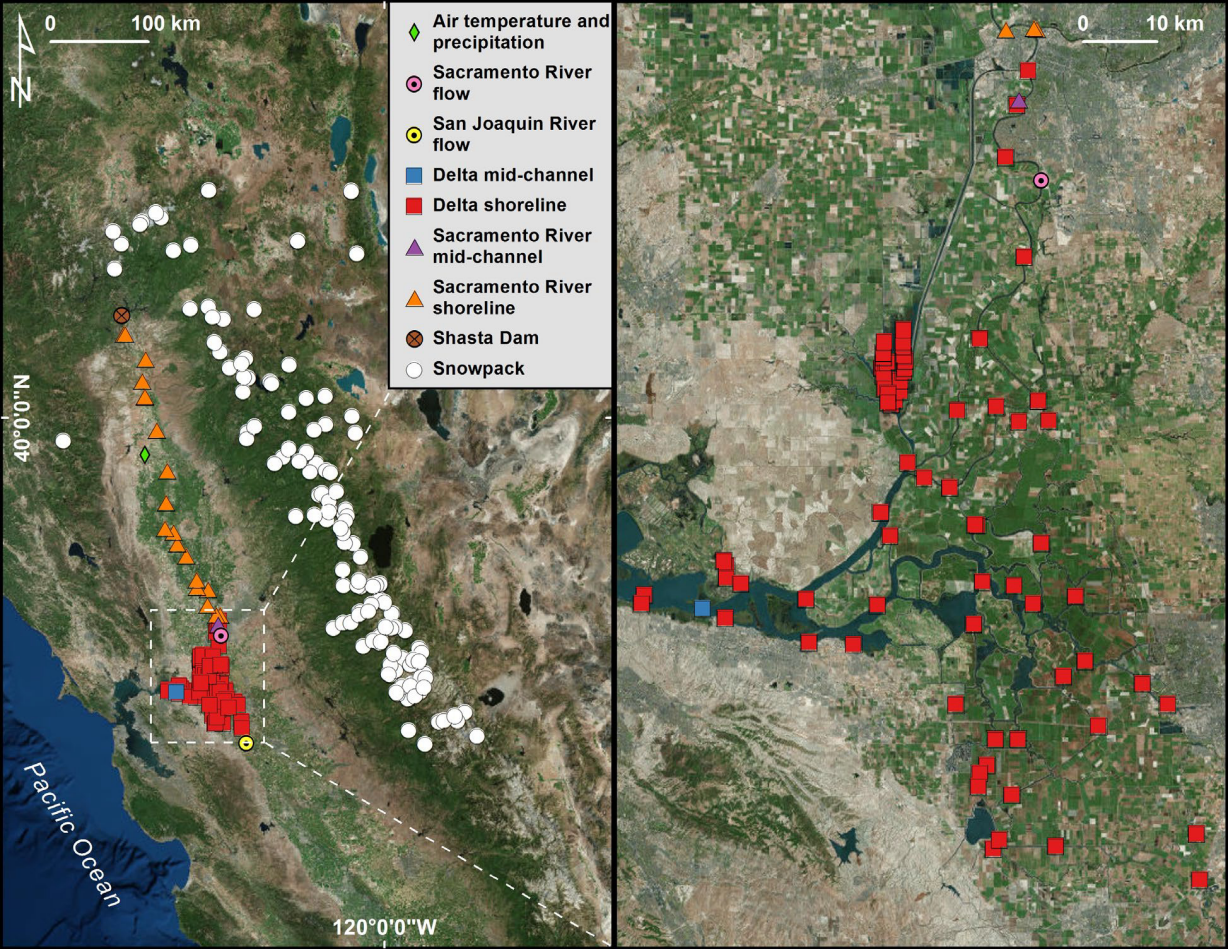
Locations within the Sacramento–San Joaquin region (California, USA) where fish presence, water temperature, air temperature, and snowpack were measured, and where Shasta Dam is located. Fish presence and water temperature were measured on‐site at near and offshore locations.

The Sacramento and San Joaquin Rivers meet in the Central Valley of California (Fig. 1). Water flows from Coast Range and Sierra Nevada headwaters into the rivers, through an extensive, now channelized, tidal Delta, and then into San Francisco Bay. Our study examined the lower Sacramento River and the Delta where water temperatures vary seasonally from 5°C to 25°C, and salinity levels are 0–0.5 ppt in the Sacramento River and 0–5 ppt in the Delta. This system experiences a Mediterranean climate, which is characterized by cool, wet winters and warm, dry summers. The Sacramento–San Joaquin watershed receives ≈ 30–40 km^3^ of rain and snow, and ≈ 40% of this annual amount is released after 1 April as snowmelt (Knowles and Cayan 2002). This water is managed via some of the world's most extensive and integrated dams, reservoirs, aqueducts, and canals to support competing interests of people (e.g., agriculture) and fish. A major component of this system's infrastructure is Shasta Dam and its reservoir, Shasta Lake, located in the Northern Sacramento Valley. Shasta Dam is by far the largest reservoir in the state and is fed by rain and snowmelt runoff. In the spring, a thermocline forms and managers release warmer waters from higher elevations of the reservoir. This allows them to preserve a deeper “cold pool” that they can later use to provide cold water during warmer months (July–October) to maintain downstream temperatures appropriate for fish spawning and rearing habitat (Danner et al. 2012). Waters are then diverted to meet intense demands of agricultural, municipal, and industrial purposes. Thus, watershed conditions are ultimately constrained by water stored in mountain snowpack and artificial reservoirs, and the water quality experienced by fish in the lower watershed is now a product of intensive hydroregulation.

We examined the phenology of juvenile Chinook salmon (*Oncorhynchus tshawytscha*), an anadromous species that rears in steams, floodplains, and estuaries of the Pacific Rim (Quinn 2005). The Sacramento River is inhabited by the Central Valley fall and late fall run, Central Valley spring run, and Sacramento winter run evolutionarily significant units, which are classified under the U.S. Endangered Species Act as species of concern, threatened, and endangered, respectively. Spring and winter run life histories in the Central Valley have declined precipitously as dams prevented fish from spawning and rearing in elevated, cooler waters (Myers et al. 1998, Yoshiyama et al. 1998). Hatcheries contribute substantially to Chinook salmon in this system, specifically to fry before 1999 and fry and smolts throughout the study window (Huber and Carlson 2015). We were initially concerned that hatchery practices (e.g., timing and size of fish released) may create artificial trends of fish responses in relation to springtime conditions, but we found little evidence that hatchery practices varied with springtime conditions (Appendix S1). Hatchery fish, however, were certainly among those observed. Chinook salmon in the Central Valley inhabit the southernmost extent of their species’ range and prefer water temperatures of 12°–15°C, but temperatures often exceed 22°C. In this system, juvenile rearing peaks February–March and outmigration peaks April–June, which is two to three months earlier than in more northern estuaries (Kjelson et al. 1982). Additionally, while other populations often include life histories that rear in fresh waters over the summer, the overwhelming majority of juveniles in the Central Valley migrate to sea as subyearlings, and often as fry, apparently to avoid warmer waters (Myers et al. 1998). Indeed, that fish are restricted by dams to lower, warmer portions of the watershed has probably decreased the expression of life history types that rear for a year before migrating to sea, and we may therefore expect that effects of temperature on phenology are especially evident in the current population compared to the historical, undeveloped system. In addition, the construction of Shasta Lake and its effect of thermal inertia on water stored from winter has cooled downstream conditions in the springtime (Boles et al. 1988); thus, historical conditions in the lower watershed, to which fish are now restricted, were probably more severe than they are currently and were always a major constraint to habitat use. Overall, summertime water temperatures constrain habitat use in our focal species and effects of temperature on the Chinook salmon population may be especially apparent in the system's current state (Kjelson et al. 1982, Myers et al. 1998).

We quantified environmental conditions and juvenile salmon responses separately across two regions and habitat types (Fig. 1). This allowed us to examine habitat use across a major portion of the region's lower watershed and compartmentalize analyses within places where the environment and fish timing were likely to be similar. We focused on two regions: the Sacramento River and the Delta. Within each region, we examined two habitat types: shoreline and mid‐channel waters. We described the timing of juvenile salmon separately for each habitat type and region because fish must encounter the river before the delta, and they often use deeper, mid‐channel waters later in the year as they grow (sensu Munsch et al. 2016). Finally, we examined the size of fish captured adjacent to Chipps Island in the mid‐channel of the Delta because this is where juvenile salmon entered the marine waters of San Francisco Bay and the Pacific Ocean beyond. That is, juvenile salmon captured at this location provide our best estimate of salmon outmigration sizes.

### Data collection

We assembled data to examine relationships among winter weather and springtime conditions of reservoirs, fish habitats, and fish responses (Fig. 1).

Data describing monthly mean air temperatures and precipitation were provided by a NOAA weather station near the Sacramento River (data *available online*).^1^ We used data describing annual snowpack archived by the California Department of Water Resources within the boundaries of the Sacramento–San Joaquin ecoregion (Abell et al. 2008) and above 36.78° N to quantify the amount of snow available to melt into the watershed (data *available online*).^2^ Snow was described by the conventional metric 1 April snow water equivalent, which is the quantity of liquid water in the snow and representative of the previous winter's snowfall because, typically, further snowfall and prior snowmelt that year are minimized.

Data describing water temperature profiles in Shasta Lake were provided by the U.S. Bureau of Reclamation. These measurements are collected at incremental depths to create a depth profile of water temperature. Because there were gaps among years in data describing temperature profiles of Shasta Lake, but these data as well as weather and snow data were often collected concurrently as far back as 1946, we used all available data describing Shasta Lake temperatures, snow, and weather dating back to 1946 to increase our power in detecting relationships among these variables.

Data describing daily water flow were provided by U.S. Geological Survey gages on the Sacramento and San Joaquin Rivers to quantify the magnitude of annual high flow events (data *available online*).^3^^,^ 4 We summed daily flow values from the two rivers to estimate flow into the Delta.

Data describing fish habitat temperature and fish presence were provided by the U.S. Fish and Wildlife Service. The Service concurrently monitors water temperatures and juvenile salmon throughout the Sacramento River and Delta via point measurements (data *available online*).^5^ That is, researchers visit many sites where they concurrently sample fish and measure water temperature. The Service repeatedly samples shorelines in many locations whereas they sample mid‐channel surface waters in two locations, each at the downstream boundary of their respective regions (Sacramento River and Delta). Along shorelines, a net is deployed parallel to shore and pulled landward to catch juvenile salmon close to shore (Brandes and McLain 2001). In the mid‐channel, a net is deployed in the channel of a flowing river to catch juvenile salmon in the middle of the river. During each netting event, researchers also measure water temperature on‐site. Shorelines are primarily inhabited by salmon fry, a life stage that occurs shortly after fish hatch and are 40–55 mm in length. Channel areas are primarily inhabited by smolts, a life stage that occurs at larger sizes as fish physiologically prepared to enter the ocean. On average, the U.S. Fish and Wildlife Service conducted 541 beach seines in the Sacramento River, 1,128 beach seines in the Delta, 1,484 trawls in the Sacramento river, and 1,806 trawls in the Delta distributed approximately evenly across every year. We examined 1992–2016 and 1995–2016 for shoreline and mid‐channel waters, respectively, because during these time periods concurrent data for all variables were available.

### Analysis

**Figure 2:**
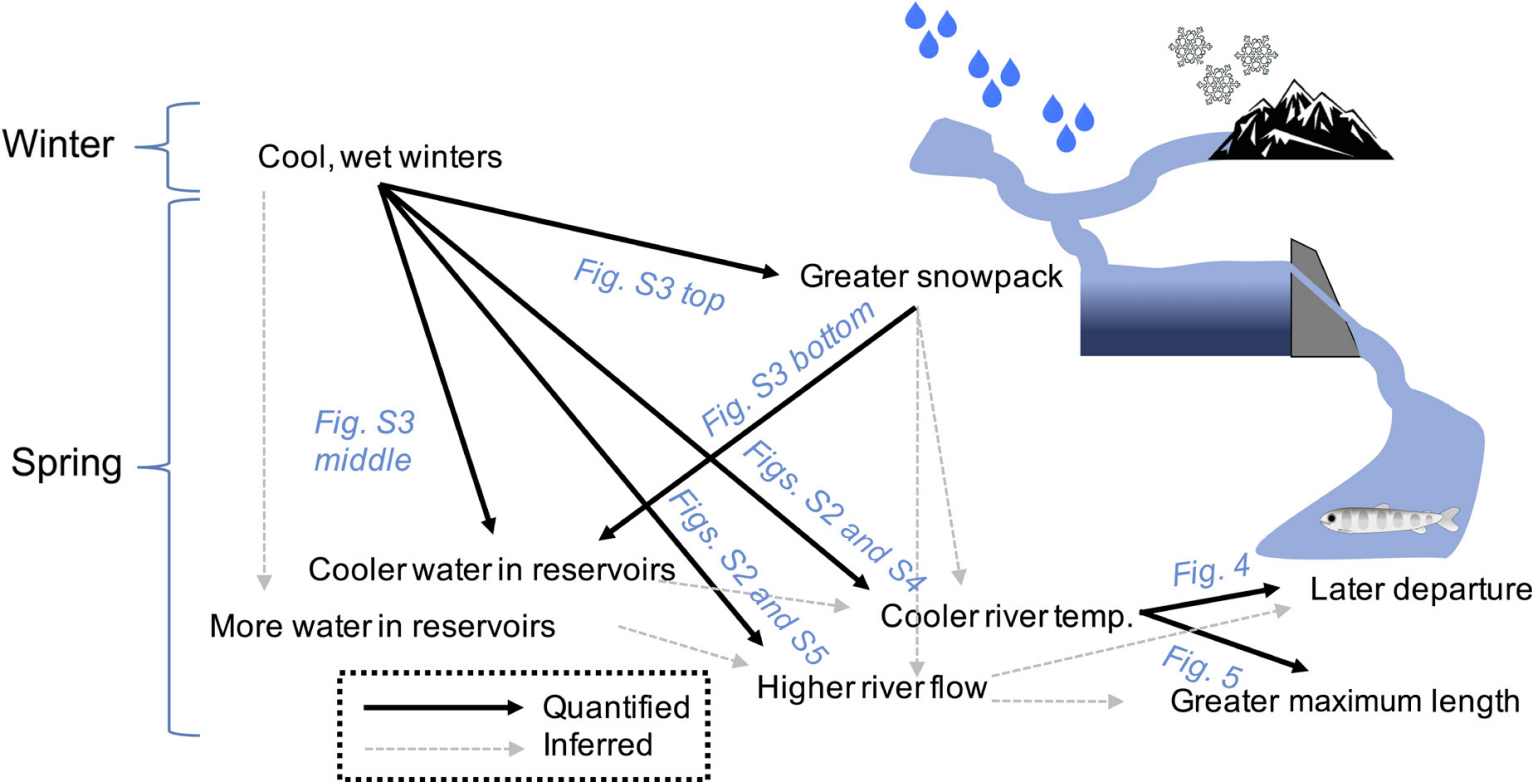
Conceptual description of our analyses. Water quantities and temperatures are deposited into reservoirs and made available downstream in the springtime according to winter precipitation and air temperature. Fish downstream respond to water conditions. Arrows indicate the influence of one factor on another factor. Supplemental figures citations refer to Appendix S2.

We quantified relationships among regional winter weather conditions, springtime snow and reservoir conditions, springtime habitat conditions, and annual fish responses (Fig. 2). Our approach was to use statistical models to convert rich data sets of environmental conditions into annual indices and then compare these indices to annual timing and maximum sizes of fish. We described model parameters used to calculate indices in the text below and listed them in Table 1 for clarity. Our analyses examined (1) water conditions in April because preliminary explorations suggested that during this month, (a) flow and temperature varied substantially among years and (b) juveniles often left the system, and (2) weather conditions during the preceding October–March because this coincided with the wet, cold season when snowpack and waters in artificial reservoirs accumulate. For brevity, we refer to October–March as winter.

**Table 1:** Parameters used in models to calculate annual indices of winter and springtime conditions

Model no.	Response	Parameters	Parameter types	Notes
1	Oct–Mar air temperature	Year + Month	Year, categorical; Month, random walk of order 2	“Year” is the annual index of winter air temperature. That is, the temperature of a given winter relative to other winters while accounting for nonlinear seasonality from Oct to Mar in temperature
2	Oct–Mar precipitation	Year + Month	Year, categorical; Month, random walk of order 2	“Year” is the annual index of winter precipitation. That is, the precipitation during a given winter relative to other winters while accounting for nonlinear seasonality from Oct to Mar in precipitation
3	log_10_(Snowpack + 1)	Year + Elevation + Station + Space	Year, categorical; Elevation, linear; Station, independent and identically distributed; Space, Gaussian Markov Random Field (Rue et al. 2009)	“Year” is the annual index of springtime snowpack. That is, the amount of snow in the mountains for a given year relative to other years while accounting for greater snowpack at higher elevations and the premise that snowpack values will be similar among observations repeated over time at the same stations and in spatially proximate stations
4	Shasta Lake surface water temperature (i.e., top 20% of water column)	Year + Depth	Year, categorical; Depth, linear	“Year” is the annual index of springtime Shasta Lake surface water temperature. That is, the temperature of surface waters for a given year relative to other years while accounting for cooler waters occurring deeper due to the thermocline
5	April water temperature (Sacramento River shoreline)	Year + Day of Year + Station	Year, categorical; Day of Year, linear; Station, independent and identically distributed	“Year” is the annual index of April water temperature. That is, the temperature of waters in April for a given year relative to other years while accounting for rising temperatures as dates approach summer and the premise that temperature values will be similar among observations repeated at the same stations over time
6	April water temperature (Delta shoreline)	Year + Day of Year + Distance to Sacramento River Main stem + Distance from San Francisco Bay + Station	Year, categorical; Day of Year, linear; Distance to Sacramento River Main stem, linear; Distance from San Francisco Bay, linear; Station, independent and identically distributed	“Year” is the annual index of April water temperature. That is, the temperature of waters in April for a given year relative to other years while accounting for rising temperatures as dates approach summer, cooler waters on the river's main stem and upstream, and the premise that temperature values will be similar among observations repeated at the same stations over time
7 and 8	April water temperature (Sacramento River and Delta mid‐channels)	Year + Day of Year	Year, categorical; Day of Year, linear	“Year” is the annual index of April water temperature. That is, the temperature of waters in April for a given year relative to other years while accounting for rising temperatures as dates approach summer

We used weather station data to quantify an annual index of air temperature and precipitation from October to March (Models 1 and 2, Table 1). In these models, the response variable was monthly temperature or precipitation and the explanatory variables were the year parameterized as a categorical variable to generate an index value and month parameterized as a random walk of the second order to account for nonlinear trends in weather as years progressed from October to March. These and all subsequent models that generated annual indices were fit to Gaussian likelihood distributions using a Bayesian approach and vague priors.

We used snowpack data to quantify an index for water content of snow in regional mountains (Model 3, Table 1). In this model, the response variable was snowpack, which was log‐transformed to normalize its distribution, and the explanatory variables were year parameterized as a categorical variable to generate an index value, elevation to account for the premise that snow is deeper at higher elevations, station (i.e., a unique sampling location) parameterized as an independent and identically distributed variable to account for non‐independence of measurements repeated at the same locations attributable to factors not explicitly included in our model, and a spatial field describing the proximity of locations to one another, which accounted for our expectation that proximate measurements will be similar due to factors not explicitly addressed by our model.

We used temperature profile data to quantify an index for each water year of temperature of waters at the surface of Shasta Lake (Model 4, Table 1). We were interested in surface water temperatures because managers release these warmers waters during the early spring so that they can conserve the cooler, deeper waters for releases during warmer portions of the year (Bartholow et al. 2001). We defined surface waters as those in the top 20% of the water column. For each year, we summarized the temperature profile at Shasta Lake by taking the median of temperatures collected at various elevations during the time period one week before and after April 1 to coincide measurements with those of snowpack and the annual time period when fish appeared to begin responding to temperature downstream. In this model, the response variable was water temperature and the explanatory variables were year parameterized as a categorical variable to generate an index value and depth to account for the premise that deeper waters will be cooler.

We used flow gauge data to quantify water flows during April for each water year. We described flow simply as the log‐transformed median daily flow for that month. This was appropriate because there were no consistent trends among years between flow in April and day of year (i.e., flow could be increasing or decreasing through April depending on the year), and we applied a log‐transformation to normalize the distribution of these data.

We used temperature data collected during beach seining and trawling to quantify for each water year indexes of temperatures during April in the Sacramento River and Sacramento–San Joaquin Delta (Models 5–8, Table 1). In these models, the response variable was water temperature and the explanatory variables were year parameterized as a categorical variable to generate an index value, day of year to account for increasing temperatures as days progressed in April and, for data collected at many stations along shorelines, station parameterized as an independent and identically distributed variable to account for non‐independence of measurements repeated at the same locations attributable to factors not explicitly included in our model. For the model describing water in the Delta, we also included variables describing the distance of measurements from the Sacramento River main stem and San Francisco Bay because preliminary data explorations suggested that waters were cooler farther upstream and on the main stem, consistent with the Sacramento River delivering cool water to the Delta. We compared indexes describing snow, weather, and water conditions via linear models to examine whether we could detect an influence of winter precipitation and temperature, as measured by the weather station, on regional snowpack (Model 9, Table 2), water temperatures near the surface of Shasta Lake (Model 10, Table 2), flow in the Sacramento River and Delta (Models 11 and 12, Table 2), and April water temperature in the shoreline and mid‐channel waters of the Sacramento River and Delta (Models 13–16, Table 2).

**Table 2:** Parameter estimates of linear models comparing winter conditions, springtime conditions, and fish responses

Model number, response, and parameter	Estimate	SE	*P*	Random effect SD
9, Springtime snowpack index				
Intercept	0.469	0.062	<0.001	
Winter precipitation index	0.002	0.000	<0.001	
Winter air temperature index	−0.029	0.005	<0.001	
10, Springtime Shasta Lake surface water temperature				
Intercept	2.330	2.555	0.369	
Winter precipitation index	−0.028	0.013	0.031	
Winter air temperature index	0.323	0.219	0.152	
11, log_10_(median Apr water flow; Sac. R.)				
Intercept	0.173	0.023	<0.001	
Winter precipitation index	0.000	0.000	0.051	
Winter air temperature index	−0.005	0.002	0.016	
12, log_10_(median Apr. water flow; Delta)				
Intercept	0.182	0.024	<0.001	
Winter precipitation index	0.000	0.000	0.041	
Winter air temperature index	−0.005	0.002	0.011	
13, Apr water temp index (Sac. R. shoreline)				
Intercept	−7.267	4.241	0.101	
Winter precipitation index	−0.029	0.016	0.090	
Winter air temperature index	1.171	0.340	0.002	
14, Apr water temp index (Delta shoreline)				
Intercept	−6.076	3.473	0.094	
Winter precipitation index	−0.019	0.013	0.160	
Winter air temperature index	1.046	0.278	0.001	
15, Apr water temperature index (Sac. R. mid‐ channel)				
Intercept	−10.886	4.738	0.033	
Winter precipitation index	−0.022	0.019	0.255	
Winter air temperature index	1.430	0.377	0.001	
16, Apr water temp index (Delta mid‐channel)				
Intercept	−6.337	2.664	0.028	
Winter precipitation index	0.000	0.011	0.963	
Winter air temperature index	1.022	0.212	<0.001	
17, Departure (Sac. R. shoreline)				
Intercept	167.508	9.426	<0.001	
Apr water temperature index	−7.278	1.554	<0.001	
18, Departure (Delta shoreline)				
Intercept	172.553	8.832	<0.001	
Apr water temperature index	−6.469	1.483	<0.001	
19, Departure (Sac. R. mid‐channel)				
Intercept	142.255	5.573	<0.001	
Apr water temperature index	−4.131	0.950	<0.001	
20, Departure (Delta mid‐channel)				
Intercept	177.274	11.702	<0.001	
Apr water temperature index	−6.341	1.939	0.004	
21, Daily max. length entering marine waters (cm)				
Intercept	12.564	0.563	<0.001	
Apr. water temp index	−0.214	0.082	0.016	
Day of year	−0.017	0.002	<0.001	
Year				0.354
log10 (Daily no. salmon measured)	(offset = 1)			

*Note:* Sac. R., Sacramento River.

Next, we described the annual timing of juvenile salmon so that we could relate timing to environmental conditions. For juvenile salmon along the shoreline, we defined annual arrivals and departures as the 5th and 95th percentile days of the year that juvenile salmon were observed for that water year. For fish in the mid‐channel, we used the same definition for arrivals, but defined departures as the 75th percentile days of the year that fish were observed. This was because annual observation dates of these fish were right‐skewed and thus percentiles describing the tail end of annual distributions (e.g., 95th percentile) were often heavily influenced by smaller numbers of migrants observed late in the summer. We combined all measurements taken alongshore of each region (i.e., the Sacramento River or Delta) whereas these conditions in mid‐channel waters were described at one location; thus, habitat conditions and fish responses along shorelines were summarized from spatially aggregated data describing a region and in mid‐channel waters they described a single station where fish were presumably leaving these regions.

In models describing the effect of springtime conditions on departure timing, the response variable was departure date and the explanatory variable was April water temperature index (Models 17–20, Table 2). We initially considered relating juvenile salmon responses to temperature *and* water flow, but these variables confounded models because they were correlated (*r* ^2^ = 0.73 [Sacramento River (Sac. R.) shore], 0.70 [Delta shore], 0.70 [Sac. R. mid‐channel], 0.38 [Delta mid‐channel]). We therefore modeled juvenile salmon responses to temperature alone, as temperature is particularly well known to impact salmon in this system (e.g., Kjelson et al. 1982), and acknowledged that flow is also an important habitat attribute and that fish likely responded to both flow and temperature.

Finally, we described the effect of springtime conditions on maximum size of juvenile salmon entering marine waters (Model 21, Table 2). In this model, we examined the size of the largest salmon observed daily at the Delta mid‐channel station (i.e., adjacent to Chipps Island) between April and August. We excluded data from days where fewer than 10 fish were observed and rare (0.22%) observations of fish above 20 cm that were probably of older age classes. During the summer, the maximum size of emigrating juveniles decreases, presumably because life histories that are timed later in the calendar year provide juveniles with less time to rear before temperatures exceed tolerances, and we therefore accounted for day of year when describing maximum size. We used a mixed effects model to describe effects of springtime conditions on maximum size (Bates et al. 2015). In this model, the response variable was the largest fish observed daily, and we parametrized (1) April water temperature index and (2) day of year as fixed effects, (3) year as a random effect to account for the premise that salmon lengths were similar within years, and (4) log‐transformed number of fish observed daily as an offset to account for the premise that larger fish were more likely to be observed on days when more total fish were observed.

We ran analyses in R version 3.3.3 (R Core Team 2019) using the packages INLA (Rue et al. 2009), lme4 (Bates et al. 2015), and ppcor (Kim 2015). We used the Bayesian package INLA to calculate indices because it allowed us to incorporate all requisite model parameters (e.g., spatial fields that accounted for spatial autocorrelation in snowpack measurements), and we used frequentist approaches to quantify linear relationships (e.g., departure timing) so we could report correlations, partial correlations, and *P* values.

## Results

**Figure 3:**
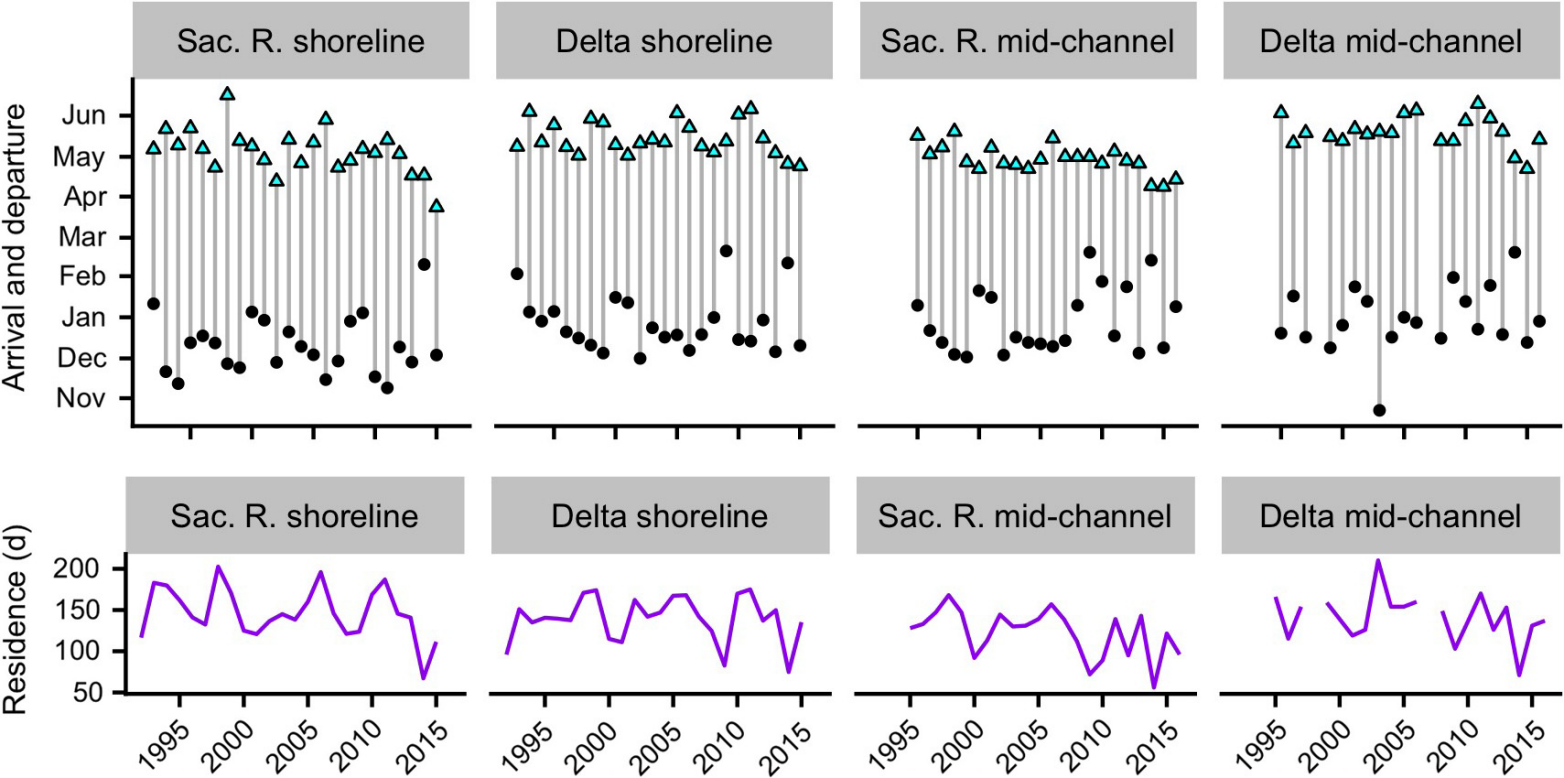
Time series of arrival (black points) and departure (cyan points) dates and total residence periods (purple lines). Residence periods are calculated by subtracting arrival dates from departure dates. Sac. R., Sacramento River.

Chinook salmon arrived in shoreline and mid‐channel waters of the Sacramento River and the Delta between November and February (Fig. 3). During their early residence in the winter, fish generally experienced cool, flowing waters (Appendix S2: Figs. S1, S2).

As winters progressed to spring, flows dropped, temperatures rose, and fish along shore increasingly occupied the coolest available waters (Appendix S2: Figs. S1, S2). Along both the upper Sacramento River and Delta shorelines, fish began using disproportionately cool waters after average temperatures of all waters (occupied and unoccupied by juvenile salmon) exceeded approximately 15°C. Across all years, this tended to occur in April.

The springtime environment experienced by fish varied substantially among years and depended on winter weather. Years with cool, wet winters left deep springtime snowpack reservoirs in the mountains (Appendix S2: Fig. S3, top) and years with wet winters produced cool springtime surface waters at Shasta Lake (Appendix S2: Fig. S3, middle). In addition, years that produced greater mountain snowpack also produced cooler surface waters at Shasta Lake (Appendix S2: Fig. S3, bottom). Cool waters in the Sacramento River and Delta persisted longer into spring if the winter was also cool (Appendix S2: Fig. S4, right). Depending on the region and habitat type, springtime waters were 3.75°–7.0°C cooler in the coolest years compared to the warmest years. While springtime waters tended to be cooler in years with greater winter precipitation, this relationship was not statistically significant (Appendix S2: Fig. S4, left). In years with cool, wet winters, springtime flows in the Sacramento River and Delta were higher (Appendix S2: Fig. S5).

**Figure 4:**
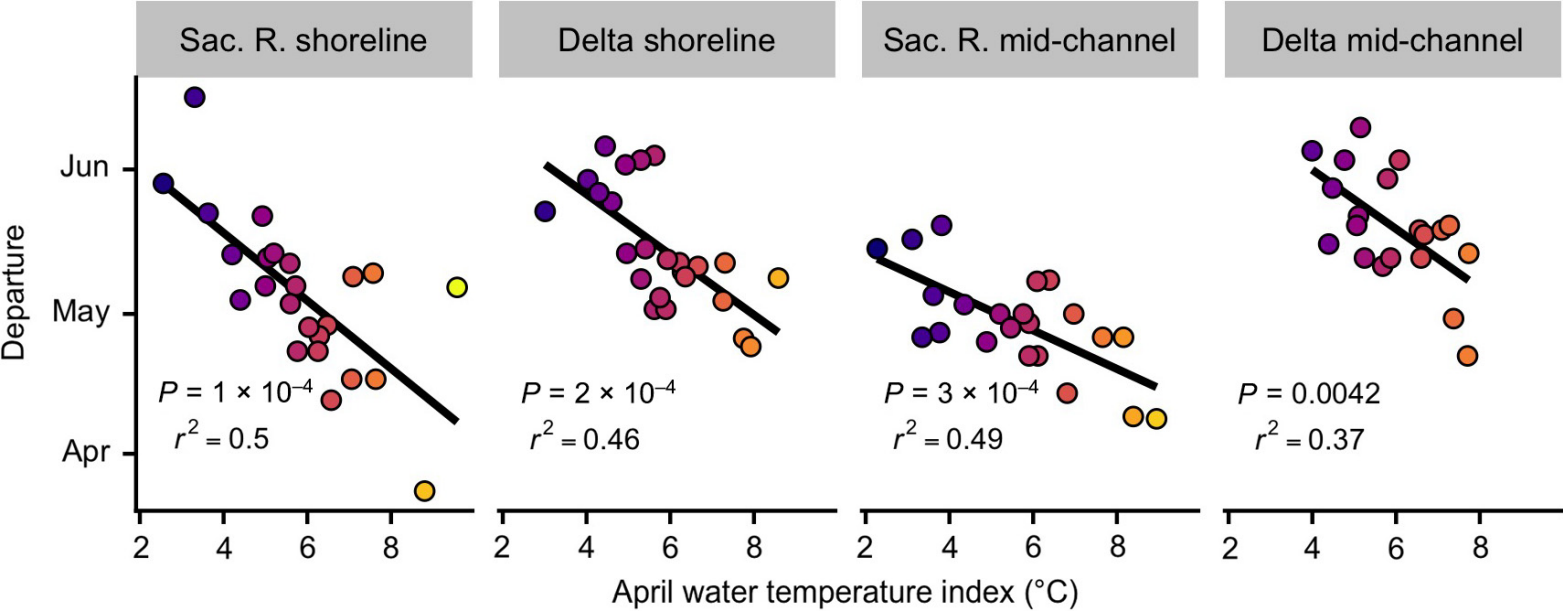
Juvenile salmon departure timing compared to April water temperature. Lines indicate relationships predicted by linear models for variables shown on the *x*‐ and *y*‐axes. Point colors correspond to April water temperature. We report correlations and *P*‐values for relationships between departure timing and April water temperature.

**Figure 5:**
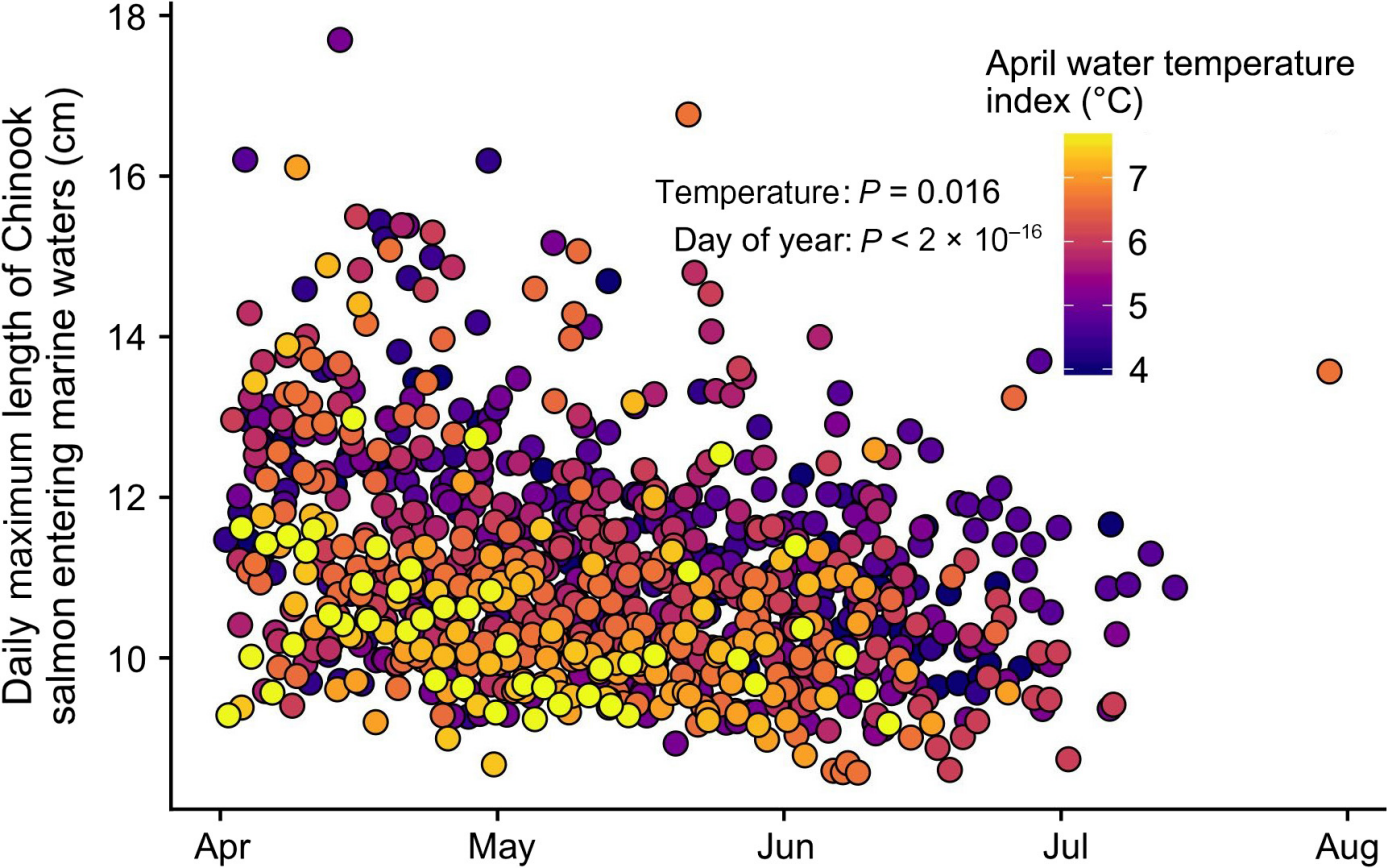
Daily maximum size of juvenile salmon entering marine waters from April to August colored by April water temperature. We report *P* values for the relationship of daily maximum size with April water temperature and date.

Warm springs advanced juvenile salmon departures and reduced their maximum sizes entering the ocean. Fish departed earlier when April water temperatures were higher (Fig. 4). Models indicated that, depending on region and habitat type, a 1°C increase in April water temperatures corresponded to fish departing four–seven days earlier (Models 17–20, Table 2). Given the range of springtime water temperatures and respective effects of water temperatures on departure, this corresponded to salmon departing the Sacramento River shoreline, Delta shoreline, Sacramento River mid‐channel, and Delta mid‐channel waters 51, 36, 28, and 24 d earlier, respectively, in the warmest years compared to the coolest years. Salmon did not depart earlier in years that they arrived earlier (correlations between arrival vs. departure date: *P* > 0.19; *r* ^2^ = 0.06, 0.08, 0.05, 0.01; Sac. R. nearshore, Delta nearshore, Sac. R. mid‐channel, Delta mid‐channel, respectively). There was a frontier of maximum lengths in salmon emigrating to sea given the date, and this frontier contracted to exclude larger fish in years with warmer springtime waters (Fig. 5). Maximum emigration sizes, given the date, decreased 0.214 cm for every 1°C increase in springtime water temperature (Fig. 5, Model 21, Table 2). This corresponded to salmon outmigrating at 0.801 cm smaller maximum sizes in the warmest years compared to the coolest years.

## Discussion

Cool, wet winters deposited cold water and snow into natural and artificial reservoirs. These sources supplied the lower watershed with cool water as the region warmed and dried in the Mediterranean spring. The extent of cool air and precipitation during the winter determined the persistence of cool, high‐flowing waters into the spring. Fish populations known to require cool temperatures and benefit from flowing waters inhabited the watershed if waters remained cool. When cool waters allowed fish populations to inhabit the watershed longer into the spring, individuals emigrated to sea at larger maximum sizes. We detected effects on timing in nearshore and mid‐channel waters of the lower Sacramento River and the Sacramento‐San Joaquin Delta, suggesting that fish responses were occurring across a major portion of the watershed. That arrival and departure timing were not correlated and that maximum sizes were smaller in years with warmer, drier winters suggests that, unlike the observations of other studies examining climate‐driven phenologies (e.g., Bradley et al. 1999), these fish truncated rather than shifted their timing in response to variable conditions. Overall, (1) winter air temperature and precipitation appeared to constrain springtime windows in which migratory fish could use their nursery habitats and (2) longer residence windows provided by cold, wet winters appeared to benefit fish by enabling growth opportunities before migrating to sea where survival is size selective (e.g., Sogard 1997, Woodson et al. 2013). More broadly, our findings contribute to an increasingly global recognition that climate can influence phenology, raising management concerns for species that alter their timing in response to changing climates (Stenseth and Mysterud 2002).

Early departures due to unfavorably warm waters in the spring suggest impaired fish habitats. First, fish in warm, dry years may experience immediate stress or mortality (Richter and Kolmes 2005). Smolts in the Sacramento River experience greater mortality when water temperatures are high and flows are low (Kjelson et al. 1982), and potentially premature migrations to sea arising from higher temperatures may further diminish the benefits of migration by disrupting tradeoffs related to predation risk. The hypothesized purpose of migration in anadromous fish is to trade off the relative predation risk and foraging opportunities of marine and fresh waters: fresh waters are relatively unproductive but offer safety from predators, the converse is true for marine environments, and estuaries appear to be intermediate (Quinn 2005). In theory, smaller fish gain more from predator refuge because they are more vulnerable (Sogard 1997). Furthermore, there appears to be a seasonal window for juveniles to enter the ocean to experience conditions conducive to fitness (e.g., high prey availability), which varies by date among years (Satterthwaite et al. 2014). Constraints on outmigration timing may therefore induce premature migrations when fish are small and vulnerable or before ocean conditions are favorable that year. Indeed, that predator life histories may no longer be synchronized with ephemeral prey (i.e., the match‐mismatch hypothesis) is a major management concern for species shifting their phenologies in response to changing climates: predators may feed suboptimally (sensu Satterthwaite et al. 2014) or engage in novel trophic interactions via shifting to alternative prey (Deacy et al. 2017). In addition, there are many nonnative, warm‐water predators of Chinook salmon in Central California (e.g., Demertras et al. 2017), and cool waters may diminish the presence of predators in juvenile salmon habitats or lower their metabolic rates and thus predation rates. Cool water therefore appears to benefit juvenile salmon in the spring by promoting extended growth and reduced predation risk, the very factors driving anadromy and estuarine residence. More generally, by expanding when juveniles could occupy certain habitats, cold waters potentially promoted fundamental nursery functions, including the ability to support optimally timed ontogenetic migrations, seasonal occurrence of necessary physical conditions, and the ability to optimize food/predation tradeoffs associated with migrations to sea (Sheaves et al. 2015).

Long annual extents of tolerable conditions may support life history diversity and be imperiled by a warming climate. Chinook salmon and many related species exhibit a diversity of life histories where their timing among habitats spanning rivers, lakes, estuaries, and oceans varies among individuals and populations (Quinn 2005). This benefits fish and people because salmon stabilize their composite populations by spreading their risk among many habitat experiences (Schindler et al. 2010) and minimize competition by spreading their density over time and space (Greene et al. 2010). However, life history variants that use the lower Sacramento River and Delta are constrained by the requirement to outmigrate before temperatures exceed thresholds, typically around April. This is concerning because California's winter temperatures are expected to increase by 1.7°–3.4°C and snowpack is expected to decrease by 29–89% by the end of the century (Hayhoe et al. 2004, Cayan et al. 2008). Our models suggest that an increase of 1.7°–3.4°C in winter air temperatures corresponds to a 1.97°C and 3.95°C increase in April temperature index, which corresponds to advancing departures by 8–29 d (depending on the region and habitat type) and decreasing maximum sizes given the date by 0.42–0.85 cm. As noted by Niels Bohr, “prediction is very difficult, especially about the future”; likewise, these numbers should be interpreted cautiously and to provide context, not as literal predictions of the future. Overall, in the future, waters may exceed tolerable conditions earlier in the year, life histories may be further constrained by requirements to depart the system earlier, and portfolio benefits derived from a diversity of life histories may be subsequently lost.

Managers may consider prolonging cool temperatures into springtime to allow juvenile salmon to use habitats more extensively. Recent advances in modeling allow managers to predictably alter downstream temperatures in the Sacramento River and other systems by releasing certain amounts and temperatures of water from reservoirs such as Shasta Lake (Danner et al. 2012, Pike et al. 2013, Caldwell et al. 2014). These efforts have largely focused on facilitating appropriate temperatures in the 100‐km reach below Keswick Dam for winter‐run Chinook salmon that spawn in late spring and early summer, and the incubation of their eggs in summer and early fall. Our results suggest that juvenile Chinook salmon rearing in the lower Sacramento River may also benefit from allocating cool waters at the onset of spring (cold water attributable to releases from dams equilibrate to the environment before waters reach the delta). This is in addition to studies that suggest greater flows promote juvenile fish outmigration survival by as much as fivefold in this system (Kjelson et al. 1982, Michel et al. 2015). Water allocations from dams in this region must meet many management targets related to people and fish, and the benefits of cooling waters in the spring for juveniles would need to be considered in this fuller context that considers the importance of human uses and other life history stages of salmon that are management priorities. Other methods that may reduce temperatures include re‐plumbing channelized systems to alter the distribution of cool water and planting riparian vegetation that blocks solar radiation (Beschta 1997).

Our study provides further evidence that climate constrains watershed use by Pacific salmon across many phases of its life cycle (reviewed by Crozier et al. 2008). Salmon embryos develop faster at warmer temperatures (Beacham and Murray 1990), but can perish in exceedingly warm or low‐flowing waters (Martin et al. 2017). Following emergence, juvenile survival can decrease in warm and low‐flowing conditions (Kjelson et al. 1982, Crozier and Zabel 2006). Notably, positive effects of warming climates may occur in cold‐constrained systems (e.g., southwestern Alaska); for example, if higher temperatures advance the timing of spring ice breakup and promote growth through increased prey availability and, potentially, metabolism (Schindler et al. 2005). In addition, the timing of juvenile downstream migrations can shift to earlier dates in warmer years (Achord et al. 2007). Related to these findings, our results suggest that (1) temperature can set upper limits on time windows in which populations can inhabit watersheds and (2) these smaller time windows prevent life histories that use the system later in the year from reaching larger sizes before heading to sea. Later, adults returning to spawn are also stressed by excessively warm conditions, and can advance the timing of their migrations upriver in response to long‐term changes in river temperature to avoid the lower, warmer portions of watersheds during the warmest part of the year (Quinn and Adams 1996). Finally, adults time their spawning according to stream temperature, presumably to synchronize the emergence of their juveniles with optimal rearing conditions (Beer and Anderson 2001). Salmon have some capacity to buffer climate‐driven stressors through plastic or evolutionary responses that include phenology, but this capacity is limited because adaptive timing in one habitat often competes with adaptive timing in another (Crozier et al. 2008). In our case, earlier migrations to sea may increase survival in the watershed but decrease survival in the ocean if seasonal prey are not yet abundant (Satterthwaite et al. 2014) or if earlier outmigrants are smaller and therefore at greater risk of predation (Sogard 1997). Overall, our findings and those of others suggest that climate often constrains when salmon use certain habitats and why, and it will be important to monitor how phenological responses across the life cycle translate ultimately to demographic responses (e.g., cohort survival).

Complexities should be considered in the interpretation of our results. First, we examined population‐level constraints rather than the experiences of individuals. For instance, individuals naturally predisposed (e.g., life history variants) to enter marine waters in the winter would presumably be less impacted by warm springs. Secondly, we chose broad‐scale metrics to describe our study system. Fish experienced a more nuanced, dynamic environment beyond what we could measure that depended on finer‐scale habitat conditions and fish movements. An example of this supported by our data is that fish may use shoreline waters until they exceed tolerable levels and then retreat to cooler mid‐channel waters before leaving the system entirely. In addition, metrics that described environmental conditions in certain months were probably correlated with those of proximate months and our models are probably measuring their response to both. However, that our model understanding of the environment correlated well with fish responses suggests that we have parsimoniously captured the phenomenon: cold, wet winters keep waters cool and flowing high longer, allowing fish to depart to sea later and larger. Finally, we may expect that, compared to more natural systems, our system's lack of juvenile age structure (e.g., age 1+ fish that rear at higher elevations before migrating to sea) and habitat complexity (e.g., extensive stream networks with coldwater refugia) may contribute to an especially apparent, population‐level phenological response of fish to temperature.

Our study would also be enhanced by a greater understanding of habitat use in mid‐channel waters and outcomes (e.g., mortality sources) of populations that departed earlier and smaller. In contrast to measurements of habitat use along shore, fish observations in mid‐channel waters only occurred in two locations. This likely limited our understanding of the temperatures that fish select for because temperatures are likely to vary substantially among locations in the watershed and may explain why, in contrast to habitat use in shoreline waters, we did not detect fish in mid‐channel waters using cooler than average temperatures in the spring. Understanding the demographic consequences (e.g., fry to adult survival) of reductions in outmigration windows and maximum outmigration sizes would further improve the application of this work for identifying the relative benefits of water management. For example, departure timing may reflect mortality as well as higher and earlier emigration rates in warm years and it would be informative to quantify relationships between fish size at emigration and survival or reproductive success at later life stages (sensu Woodson et al. 2013). It would be especially informative to determine how watershed habitat conditions may interact (e.g., synergistically, additively, antagonistically) with conditions experienced during nearshore and marine life stages to determine overall survival.

Migration enables many taxa to be in the right place at the right time. For juvenile Chinook salmon in the Sacramento River and Delta, the “right time” appears to be when waters are cool and flowing high. In this region, precipitation occurs mostly in winter, but mountain snowpack and artificial reservoirs store water that is released in the spring. This delays the onset of intolerably warm aquatic environments despite warming weather and increases the time window in which migratory fish can use their freshwater and estuarine habitats. The extent of habitat use for coldwater species in watershed ecosystems may therefore depend on cool, wet winters. We studied a species where it was especially responsive to low stream flows and high water temperatures, but snowmelt and air temperature are fundamental to fish habitat conditions in spring for many aquatic ecosystems. We should therefore consider that, in systems fed by snowmelt or artificial reservoirs, warm, dry winters (e.g., recent drought in California) may portend poor nursery habitat conditions for fish that year. This is significant because many species rely on freshwater and estuarine waters during critical juvenile phases (Beck et al. 2001), these fish often develop to support essential functions in marine ecosystems (Sheaves et al. 2015), and snowpack and air temperature conditions are changing worldwide (Barnett et al. 2005). Indeed, in recent years with warm, dry winters, juvenile Chinook salmon inhabited Central California briefly, which is concerning if it foreshadows warming winters and threats to life histories that migrate through the system later in the spring.

However, ecologists and managers are developing more sophisticated and nuanced approaches to water regulation in conservation contexts (Danner et al. 2012). Within constraints set by climate, regulation strategies can mitigate periods that are stressful to fish if we quantitatively understand the impacts of flow and temperature on fish performance (e.g., egg survival; Martin et al. 2017). Concerted research efforts may therefore seek to understand critical ontogenetic and annual periods when flow and temperature matter most to fish, which may allow us to develop regulatory strategies that optimize for human water needs and conservation impacts.

## Notes

1
https://www.ncdc.noaa.gov/cdo-web/datasets/GSOM/stations/GHCND:USC00046506/detail

2
https://cdec.water.ca.gov/snow/current/snow/

3
https://waterdata.usgs.gov/usa/nwis/uv?site_no=11447650

4
https://waterdata.usgs.gov/nwis/uv?site_no=11303500

5
https://www.fws.gov/lodi/juvenile_fish_monitoring_program/jfmp_index.htm

## References

[eap1880-bib-0001] Abell, R. , et al. 2008 Freshwater ecoregions of the world: a new map of biogeographic units for freshwater biodiversity conservation. BioScience 58:403–414.

[eap1880-bib-0002] Achord, S. , R. W. Zabel , and B. P. Sandford . 2007 Migration timing, growth, and estimated parr‐to‐smolt survival rates of wild Snake River spring‐summer Chinook salmon from the Salmon River Basin, Idaho, to the Lower Snake River. Transactions of the American Fisheries Society 136:142–154.

[eap1880-bib-0003] Barnett, T. P. , J. C. Adam , and D. P. Lettenmaier . 2005 Potential impacts of a warming climate on water availability in snow‐dominated regions. Nature 438:303–309. 16292301 10.1038/nature04141

[eap1880-bib-0004] Bartholow, J. , R. B. Hanna , L. Saito , D. Lieberman , and M. Horn . 2001 Simulated limnological effects of the Shasta Lake temperature control device. Environmental Management 27:609–626. 11289458 10.1007/s0026702324

[eap1880-bib-0005] Bates, D. , M. Maechler , B. M. Bolker , and S. C. Walker . 2015 Fitting linear mixed‐effects models using lme4. Journal of Statistical Software 67:1–48.

[eap1880-bib-0006] Beacham, T. D. , and C. B. Murray . 1990 Temperature, egg size, and development of embryos and alevins of five species of Pacific salmon: a comparative analysis. Transactions of the American Fisheries Society 119:927–945.

[eap1880-bib-0007] Beck, M. W. , et al. 2001 The identification, conservation, and management of estuarine and marine nurseries for fish and invertebrates: A better understanding of the habitats that serve as nurseries for marine species and the factors that create site‐specific variability in nursery quality will improve conservation and management of these areas. BioScience 51:633–641.

[eap1880-bib-0008] Beer, W. N. , and J. J. Anderson . 2001 Effect of spawning day and temperature on salmon emergence: interpretations of a growth model for Methow River Chinook. Canadian Journal of Fisheries and Aquatic Sciences 58:943–949.

[eap1880-bib-0009] Beschta, R. L. 1997 Riparian shade and stream temperature: an alternative perspective. Rangelands 19:25–28.

[eap1880-bib-0501] Boles, G. L. , S. M. Turek , C. C. Maxwell , and D. M. McGill . 1988 Water temperature effects on Chinook salmon (Oncorhynchus tshawytscha) with emphasis on the Sacramento River: a literature review. California Department of Water Resources, Sacramento, California, USA.

[eap1880-bib-0010] Bradley, N. L. , A. C. Leopold , J. Ross , and W. Huffaker . 1999 Phenological changes reflect climate change in Wisconsin. Proceedings of the National Academy of Sciences USA 96:9701–9704. 10.1073/pnas.96.17.9701 PMC22273 10449757

[eap1880-bib-0011] Brandes, P. L. , and J. S. McLain . 2001 Juvenile Chinook salmon abundance, distribution, and survival in the Sacramento‐San Joaquin Estuary. Pages 39–136 *in* Contributions to the Biology of the Central Valley Salmonids. Fish Bulletin 179: Volume 2. California Department of Fish and Game, Sacramento, California, USA.

[eap1880-bib-0012] Caldwell, J. , B. Rajagopalan , and E. Danner . 2014 Statistical modeling of daily water temperature attributes on the Sacramento River. Journal of Hydrologic Engineering 20:04014065.

[eap1880-bib-0013] Cayan, D. R. , E. P. Maurer , M. D. Dettinger , M. Tyree , and K. Hayhoe . 2008 Climate change scenarios for the California region. Climatic Change 87:21–42.

[eap1880-bib-0014] Couto, T. B. , and J. D. Olden . 2018 Global proliferation of small hydropower plants—science and policy. Frontiers in Ecology and the Environment 16:91–100.

[eap1880-bib-0015] Crozier, L. G. , and R. W. Zabel . 2006 Climate impacts at multiple scales: evidence for differential population responses in juvenile Chinook salmon. Journal of Animal Ecology 75:1100–1109. 16922845 10.1111/j.1365-2656.2006.01130.x

[eap1880-bib-0016] Crozier, L. G. , A. P. Hendry , P. W. Lawson , T. P. Quinn , N. J. Mantua , J. Battin , R. G. Shaw , and R. B. Huey . 2008 Potential responses to climate change in organisms with complex life histories: evolution and plasticity in Pacific salmon. Evolutionary Applications 1:252–270. 25567630 10.1111/j.1752-4571.2008.00033.x PMC3352429

[eap1880-bib-0017] Danner, E. M. , F. S. Melton , A. Pike , H. Hashimoto , A. Michaelis , B. Rajagopalan , J. Caldwell , L. DeWitt , S. Lindley , and R. R. Nemani . 2012 River temperature forecasting: a coupled‐modeling framework for management of river habitat. IEEE Journal of Selected Topics in Applied Earth Observations and Remote Sensing 5:1752–2760.

[eap1880-bib-0018] Deacy, W. W. , J. B. Armstrong , W. B. Leacock , C. T. Robbins , D. D. Gustine , E. J. Ward , J. A. Erlenbach , and J. A. Stanford . 2017 Phenological synchronization disrupts trophic interactions between Kodiak brown bears and salmon. Proceedings of the National Academy of Sciences USA 114:10432–10437. 10.1073/pnas.1705248114 PMC5625906 28827339

[eap1880-bib-0019] Demertras, N. J. , D. D. Huff , C. J. Michel , J. M. Smith , G. R. Cutter , S. A. Hayes , and S. T. Lindley . 2017 Development of underwater recorders to quantify predation of juvenile Chinook salmon (*Oncorhynchus tshawytscha*) in a river environment. Fishery Bulletin 114:179–185.

[eap1880-bib-0021] Greene, C. M. , J. E. Hall , K. R. Guilbault , and T. P. Quinn . 2010 Improved viability of populations with diverse life‐history portfolios. Biology Letters 6:382–386. 20007162 10.1098/rsbl.2009.0780 PMC2880035

[eap1880-bib-0022] Hayhoe, K. , et al. 2004 Emissions pathways, climate change, and impacts on California. Proceedings of the National Academy of Sciences USA 101:12422–12427. 10.1073/pnas.0404500101 PMC514653 15314227

[eap1880-bib-0023] Huber, E. R. , and S. M. Carlson . 2015 Temporal trends in hatchery releases of fall‐run Chinook salmon in California's Central Valley. San Francisco Estuary and Watershed Science 12 10.15447/sfews.2015v13iss2art3

[eap1880-bib-0024] Kim, S. 2015 ppcor: Partial and Semi‐Partial (Part) Correlation. R package version 1.1. https://CRAN.R-project.org/package=ppcor

[eap1880-bib-0025] Kjelson, M. A. , P. F. Raquel , and F. W. Fisher . 1982 Life history of fall‐run juvenile Chinook salmon, *Oncorhynchus tshawytscha*, in the Sacramento‐San Joaquin estuary, California Pages 393–411 *in* Kennedy V. S., editor. Estuarine comparisons. Academic Press, Cambridge, Massachusetts, USA.

[eap1880-bib-0026] Knowles, N. , and D. R. Cayan . 2002 Potential effects of global warming on the Sacramento/San Joaquin watershed and the San Francisco estuary. Geophysical Research Letters 29:38‐1–38‐4.

[eap1880-bib-0027] Mantua, N. J. , L. G. Crozier , T. E. Reed , D. E. Schindler , and R. S. Waples . 2015 Response of Chinook salmon to climate change. Nature Climate Change 5:613–615.

[eap1880-bib-0028] Martin, B. T. , A. Pike , S. N. John , N. Hamda , J. Roberts , S. T. Lindley , and E. M. Danner . 2017 Phenomenological vs. biophysical models of thermal stress in aquatic eggs. Ecology Letters 20:50–59. 27891770 10.1111/ele.12705

[eap1880-bib-0029] Michel, C. J. , A. J. Ammann , S. T. Lindley , P. T. Sandstrom , E. D. Chapman , M. J. Thomas , G. P. Singer , A. Peter Klimley , and R. Bruce MacFarlane . 2015 Chinook salmon outmigration survival in wet and dry years in California's Sacramento River. Canadian Journal of Fisheries and Aquatic Sciences 72:1749–1759.

[eap1880-bib-0030] Mote, P. W. , S. Li , D. P. Lettenmaier , M. Xiao , and R. Engel . 2018 Dramatic declines in snowpack in the western US. Climate and Atmospheric Science. 10.1038/s41612-018-0012-1

[eap1880-bib-0031] Munsch, S. H. , J. R. Cordell , and J. D. Toft . 2016 Fine‐scale habitat use and behavior of a nearshore fish community: nursery functions, predation avoidance, and spatiotemporal habitat partitioning. Marine Ecology Progress Series 557:1–15.

[eap1880-bib-0032] Myers, J. M. , et al. 1998 Status review of chinook salmon from Washington, Idaho, Oregon, and California. US. Dept. Commer, NOAA Tech, Memo. NMFS‐NWFSC 35, 443 p.

[eap1880-bib-0033] Nickel, D. K. , M. T. Brett , and A. D. Jassby . 2004 Factors regulating Shasta Lake (California) cold water accumulation, a resource for endangered salmon conservation. Water Resources Research. 10.1029/2003wr002669

[eap1880-bib-0034] Olden, J. D. , and R. J. Naiman . 2009 Incorporating thermal regimes into environmental flows assessments: modifying dam operations to restore freshwater ecosystem integrity. Freshwater Biology 55:86–107.

[eap1880-bib-0035] O'Reilly, C. M. , et al. 2015 Rapid and highly variable warming of lake surface waters around the globe. Geophysical Research Letters 42:10773–10781.

[eap1880-bib-0036] Pike, A. , E. Danner , D. Boughton , F. Melton , R. Nemani , B. Rajagopalan , and S. Lindley . 2013 Forecasting river temperatures in real time using a stochastic dynamics approach. Water Resources Research 49:5168–5182.

[eap1880-bib-0037] Quinn, T. P. 2005 The behavior and ecology of Pacific salmon and trout. University of Washington Press, Seattle, Washington, USA.

[eap1880-bib-0038] Quinn, T. P. , and D. J. Adams . 1996 Environmental changes affecting the migratory timing of American shad and sockeye salmon. Ecology 77:1151–1162.

[eap1880-bib-0039] R Core Team . 2019 R: a language and environment for statistical computing. R Foundation for Statistical Computing, Vienna, Austria https://www.r-project.org

[eap1880-bib-0040] Richter, A. , and S. A. Kolmes . 2005 Maximum temperature limits for Chinook, Coho, and chum salmon, and steelhead trout in the Pacific Northwest. Reviews in Fisheries Science 13:23–49.

[eap1880-bib-0041] Rue, H. , S. Martino , and N. Chopin . 2009 Approximate Bayesian inference for latent Gaussian models by using integrated nested Laplace approximations. Journal of the Royal Statistical Society: Series B (Statistical Methodology) 71:319–392.

[eap1880-bib-0042] Satterthwaite, W. H. , S. M. Carlson , S. D. Allen‐Moran , S. Vincenzi , S. J. Bograd , and B. K. Wells . 2014 Match‐mismatch dynamics and the relationship between ocean‐entry timing and relative ocean recoveries of Central Valley fall run Chinook salmon. Marine Ecology Progress Series 511:237–248.

[eap1880-bib-0043] Schindler, D. E. , D. E. Rogers , M. D. Scheuerell , and C. A. Abrey . 2005 Effects of changing climate on zooplankton and juvenile sockeye salmon growth in southwestern Alaska. Ecology 86:198–209.

[eap1880-bib-0044] Schindler, D. E. , R. Hilborn , B. Chasco , C. P. Boatright , T. P. Quinn , L. A. Rogers , and M. S. Webster . 2010 Population diversity and the portfolio effect in an exploited species. Nature 465:609–613. 20520713 10.1038/nature09060

[eap1880-bib-0045] Sheaves, M. , R. Baker , I. Nagelkerken , and R. M. Connolly . 2015 True value of estuarine and coastal nurseries for fish: incorporating complexity and dynamics. Estuaries and Coasts 38:401–414.

[eap1880-bib-0046] Sogard, S. M. 1997 Size‐selective mortality in the juvenile stage of teleost fishes: a review. Bulletin of Marine Science 60:1129–1157.

[eap1880-bib-0047] Srygley, R. B. , R. Dudley , E. G. Oliveira , R. Aizprua , N. Z. Pelaez , and A. J. Riveros . 2010 El Niño and dry season rainfall influence hostplant phenology and an annual butterfly migration from Neotropical wet to dry forests. Global Change Biology 16:936–945.

[eap1880-bib-0048] Stenseth, N. C. , and A. Mysterud . 2002 Climate, changing phenology, and other life history traits: nonlinearity and match‐mismatch to the environment. Proceedings of the National Academy of Sciences USA 21:13379–13381. 10.1073/pnas.212519399 PMC129680 12370424

[eap1880-bib-0049] Van Der Jeugd, H. P. , G. Eichhorn , K. E. Litvin , J. Stahl , K. Larsson , A. J. Van Der Graaf , and R. H. Drent . 2009 Keeping up with early springs: rapid range expansion in an avian herbivore incurs a mismatch between reproductive timing and food supply. Global Change Biology 15:1057–1071.

[eap1880-bib-0050] Woodson, L. E. , B. K. Wells , P. K. Weber , R. B. MacFarlane , G. E. Whitman , and R. C. Johnson . 2013 Size, growth, and origin‐dependent mortality of juvenile Chinook salmon *Oncorhynchus tshawytscha* during early ocean residence. Marine Ecology Progress Series 487:163–175.

[eap1880-bib-0051] Yoshiyama, R. M. , F. W. Fisher , and P. B. Moyle . 1998 Historical abundance and decline of Chinook salmon in the Central Valley region of California. North American Journal of Fisheries Management 18:487–521.

