# Supplementary material for: Warm, dry winters truncate timing and size distribution of seaward‐migrating salmon across a large, regulated watershed
Source: Ecol Appl. 2019 Apr 8;29(4):e01880. doi: 10.1002/eap.1880 (PMC6850174; doi:10.1002/eap.1880)
Supplement: Supplementary file 1 [file EAP-29-na-s001.pdf]

Supporting Information. Munsch S. H., C. M. Greene, R. C. Johnson, W. H. Satterthwaite, H. Imaki, and P. L. Brandes. 2019. Warm, dry winters truncate timing and size distribution of seaward-migrating salmon across a large, regulated watershed. *Ecological Applications.*

Appendix S1. *Examining relationships between hatchery practices and springtime water conditions*.

There are four runs of Chinook salmon in the Central Valley of California named after the timing of adults returning to the system. Given differences in the timing of spawning among runs, the timing of juveniles in the system also varies among runs, with spring-run and fall-run fish primarily inhabiting the Central Valley during our study's focus in spring and summer. Figure. S1 shows the Central Valley's conventional length at date estimations of runs, illustrating the order of juvenile salmon runs in the system. Based on these timings, we inferred that fall-run Chinook salmon were likely driving the timing of departures and spring and fall-run Chinook salmon contributed to estimates of maximum outmigration size.

Chinook salmon in the Central Valley are supported by artificial propagation programs (described in detail by Huber and Carlson 2015) and we were concerned that the timing and size of Chinook salmon in the Central Valley may be driven by the response of hatcheries to springtime temperatures rather than the behavioral response of the salmon to springtime temperatures. We therefore investigated the timing and size of hatchery releases of spring and fall-run Chinook in relation to springtime conditions. We used data describing the release of fall-run Chinook salmon from Huber and Carlson (2015) and spring-run Chinook salmon from California Department of Fish and Wildlife (https://www.wildlife.ca.gov/fishing/hatcheries) and compared it to our estimates of April water temperature indexes. These data provided release locations, allowing us to exclude from comparisons releases of fish downstream of the Sacramento River and Delta regions in our study.

First, we examined the timing of fall-run Chinook salmon, which are the predominant salmon run produced by hatcheries, in relation to April water temperature indexes (Fig. S2). Most releases occurred between April and June, with inconsistent release timings among warm and cool years toward the end of annual release windows. In particular, salmon were not consistently released earlier or in lower abundances in years with warmer springs. Exceptions to this occurred in 2014 and 2015, when waters were warm and few salmon were released above the lower boundary of our Sacramento River region. We therefore repeated our analyses on departure along the Sacramento River shoreline and in its mid-channel excluding these years. This did not change our principal findings in the main text that warmer springs advance salmon departures (Fig. S3). We also noted that (1) hatchery salmon were often released well before and after departure dates along shorelines, suggesting that the timing of hatchery releases were not driving the timing of fish observed along shore and (2) the linear trend relating departure timing to springtime temperatures occurred across the range of temperatures observed in our study window; that is, our principal findings were not driven by extreme years when hatcheries were more likely to respond to emergency scenarios such as drought.

Next, we examined the size of spring and fall-run Chinook salmon hatchery releases in relation to springtime water temperatures (Figs. S4 & S5). Fish were released across the spring and summer period and at many sizes. Examining the figures, we found little evidence that fish

were consistently released at (1) larger sizes in colder years or (2) at larger sizes given the date during colder years. There was an abundance of data describing fall-run releases, and we tested for an effect of size at date to attempt to detect subtle differences in the size of salmon released given the date. We used a mixed effects model parametrized similarly to our model in the main text that tested for an effect of spring temperature on maximum salmon size given the date. In this model, the response variable was the average fish mass at release of subyearling salmon between January and September, the fixed effects were April water temperature in the Delta mid-channel and the day of year, and the random effect was the year (Table S1). There was no significant effect of April water temperature on the size at date of hatchery salmon released.

Given the (1) lack of patterns between April water temperature and hatchery salmon release timing, maximum sizes, or sizes at given dates, (2) lack of synchrony between hatchery releases and salmon presence along shore, and (3) clear, linear patterns of April water temperature with departures and maximum sizes across the range of springtime conditions observed (i.e., the results presented in the main text), we concluded that our principal findings (salmon depart earlier and smaller in warmer years) were unlikely to be an artifact of hatchery practices.

Table S1. Summary statistics of linear mixed effects model comparing mass of hatchery salmon released to April water temperature index and day of year.

Response	Parameter	Estimate	SE	p value	Random effect SD
Mean mass of hatchery salmon released	Intercept	-2.49	1.16	0.0441	0.9297
	Apr. water temp. index	0.106	0.19	0.5828	
	Day of year	0.0713	0.000152	<2e-16	

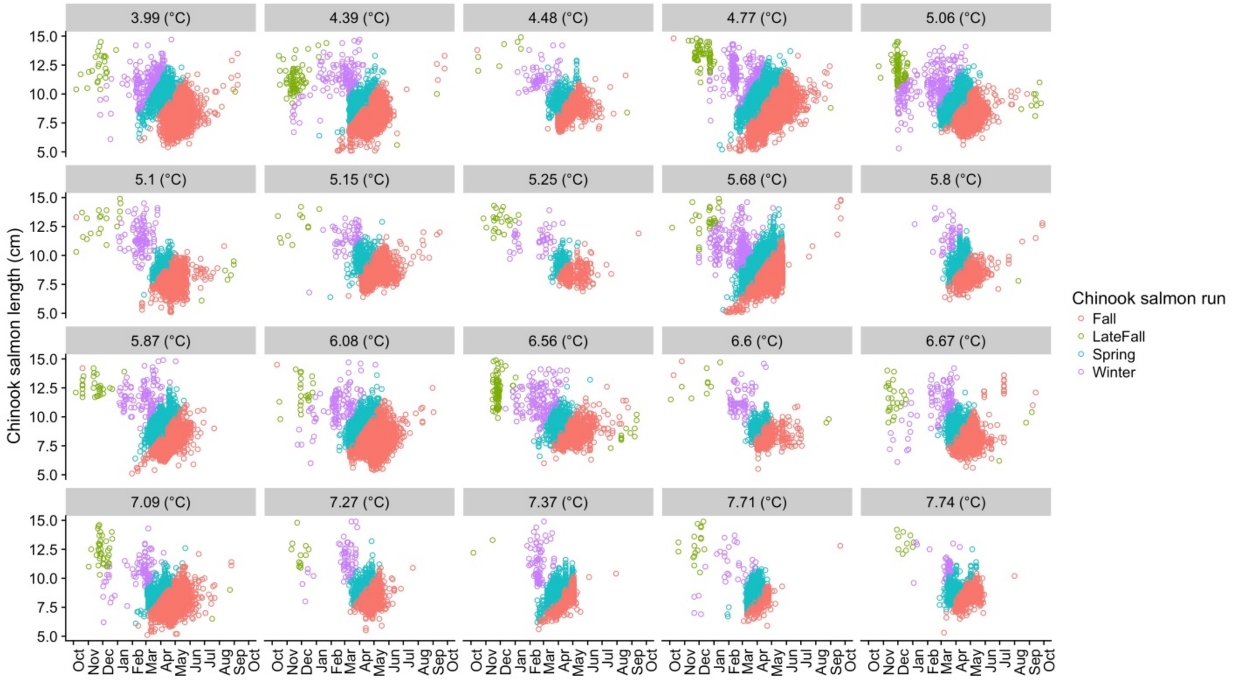

Figure S1. Length of juvenile salmon captured in the Delta mid-channel trawl compared to date, colored by Chinook salmon run, and faceted annually by April water temperature index. Chinook salmon runs are assigned by the Central Valley's length at date model, which we show to illustrate the order of salmon runs in the system.

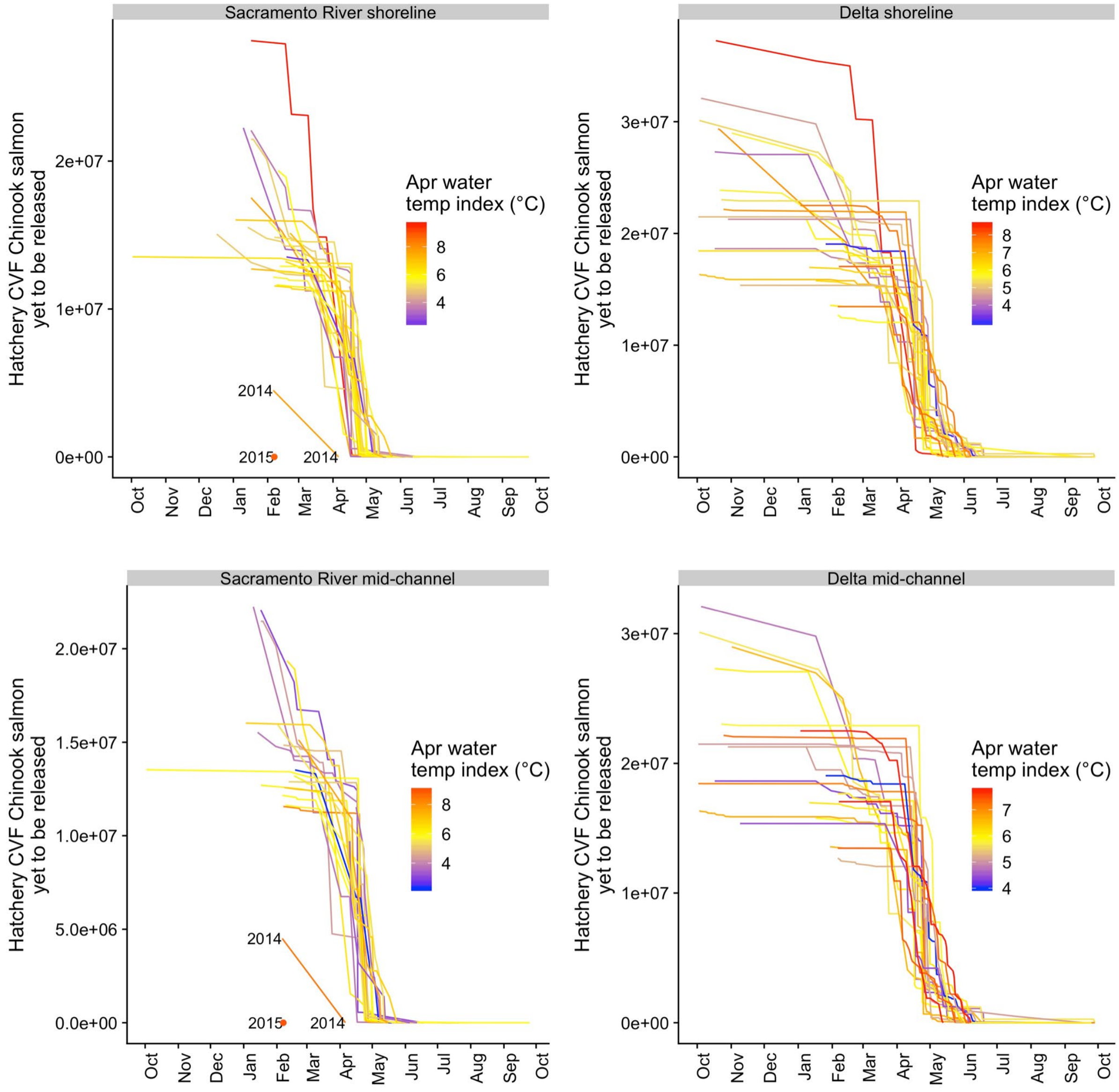

Figure S2. Reversed cumulative number (i.e., counting down the water year's total releases to zero) of hatchery Central Valley fall-run Chinook salmon released colored by springtime water temperatures. Lines show reverse cumulative numbers to highlight differences, if any, in the timing of releases at the end of the season, which would be most likely to departure estimates.

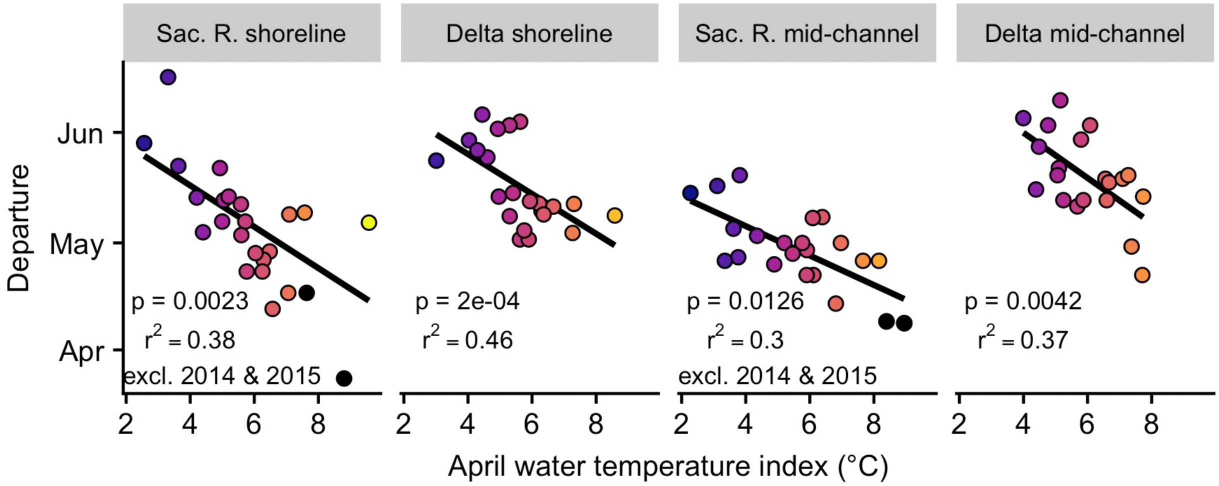

Figure S3. Departure timing of hatchery fish compared to springtime water temperatures via linear models. Linear models in the Sacramento River region are performed excluding data from 2014 and 2015 because in these years few Chinook salmon were released in this region, waters were warm, and departures were early. We report p-values and correlation coefficients.

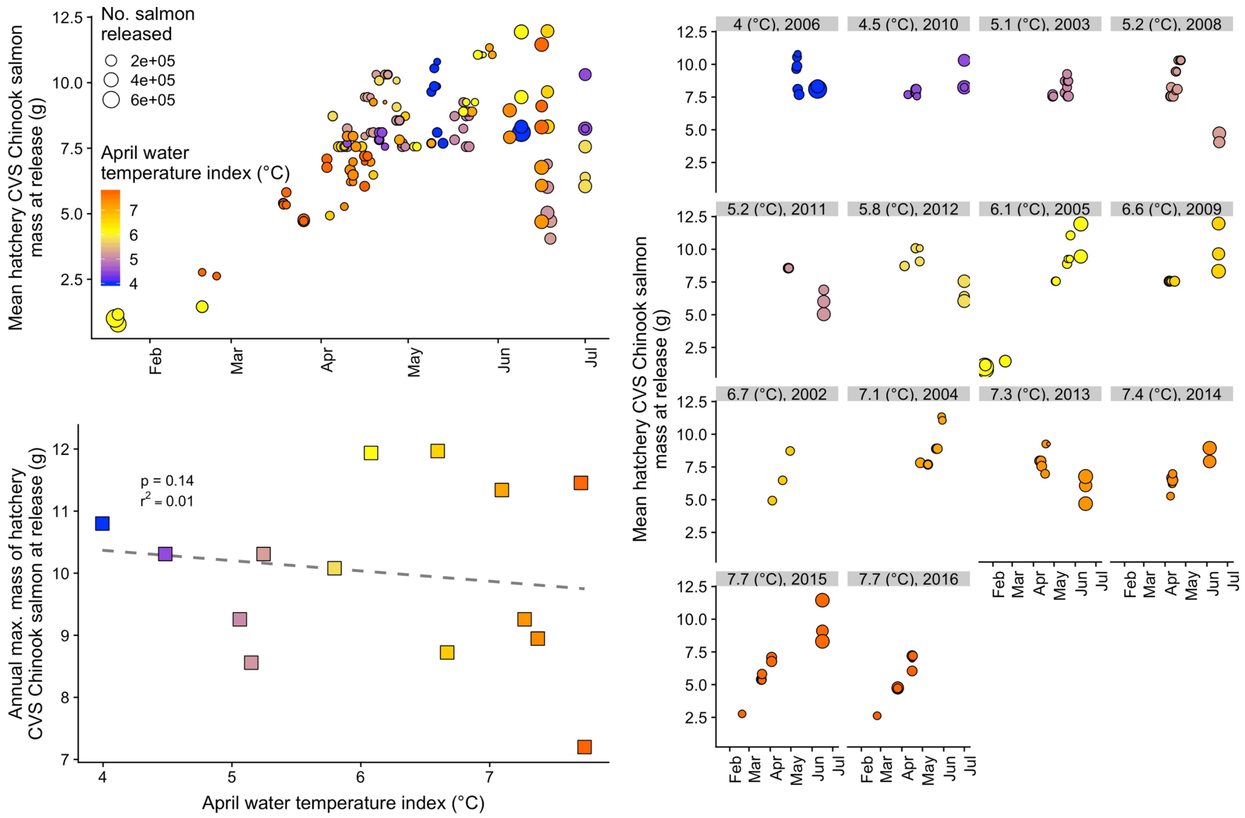

Figure S4. Timing and size of Central Valley Spring-run Chinook salmon hatchery releases. Top left: all observations combined. Right: observations seperated by year and faceted by April water temperature index. Bottom left: annual maximum release size compared via a linear model to April water temperature index. We report this relationship's p-value and correlation coefficient.

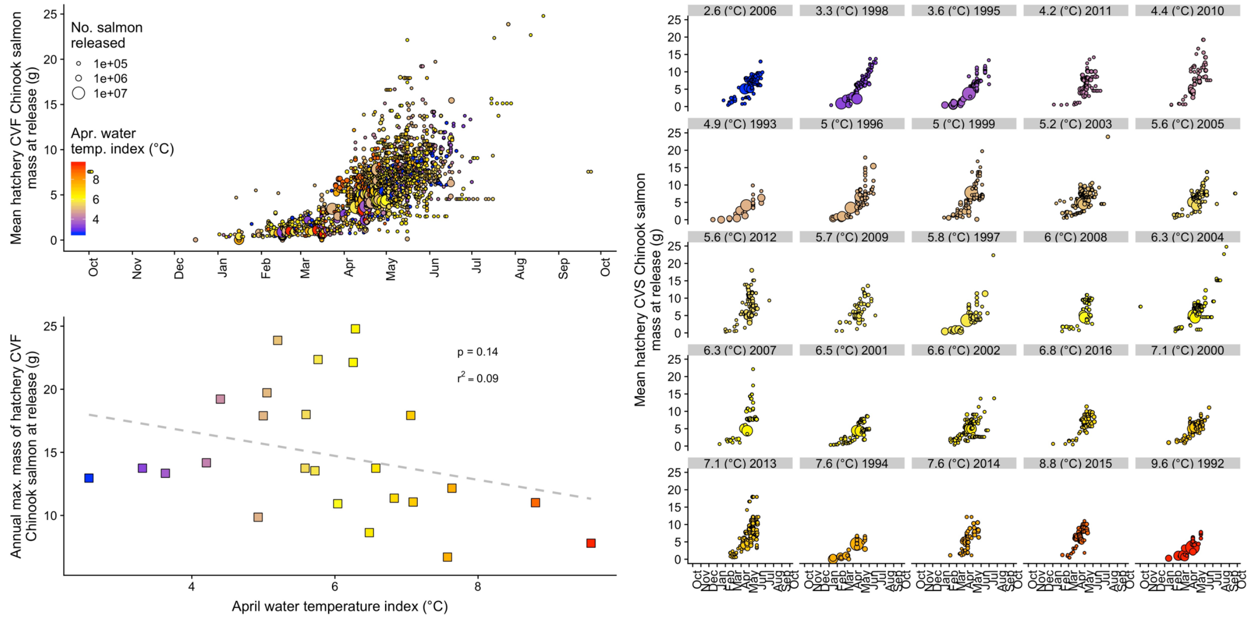

Figure S5. Timing and size of Central Valley fall-run Chinook salmon hatchery releases. Top left: all observations combined. Right: observations seperated by year and faceted by April water temperature index. Bottom left: annual maximum release size compared via a linear model to April water temperature index. We report this relationship's p-value and correlation coefficient.

Literature Cited

Huber E. R. and S. M. Carlson. 2015. Temporal trends in hatchery releases of fall-run Chinook salmon in California's Central Valley. San Francisco Estuary and Watershed Science 12
