# Supplementary material for: Warm, dry winters truncate timing and size distribution of seaward‐migrating salmon across a large, regulated watershed
Source: Ecol Appl. 2019 Apr 8;29(4):e01880. doi: 10.1002/eap.1880 (PMC6850174; doi:10.1002/eap.1880)
Supplement: Supplementary file 2 [file EAP-29-na-s002.pdf]

Supporting Information. Munsch S. H., C. M. Greene, R. C. Johnson, W. H. Satterthwaite, H. Imaki, and P. L. Brandes. 2019. Warm, dry winters truncate timing and size distribution of seaward-migrating salmon across a large, regulated watershed. *Ecological Applications.*

Appendix S2. *Supplemental figures describing relationships among environmental conditions*

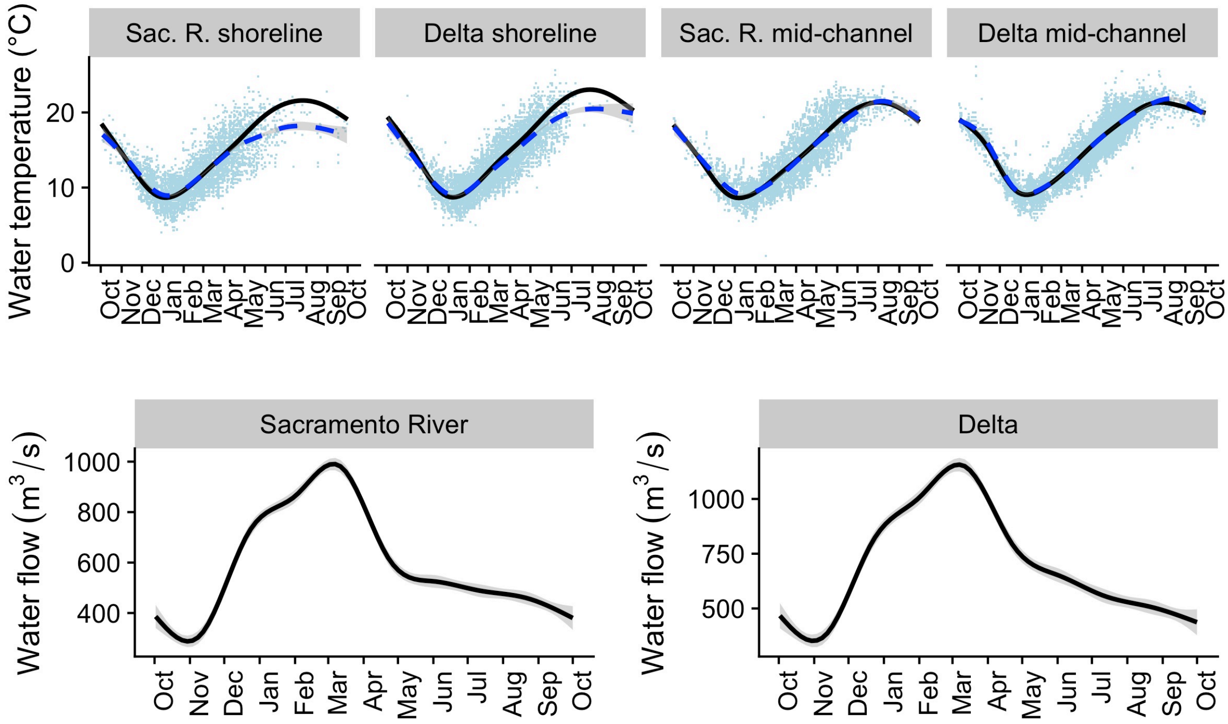

Figure S1. Top: seasonal patterns in water temperature comparing temperatures with fish present (dashed blue line) to all sampled water temperatures (solid black line). Light blue points indicate individual sampling events where fish were present to describe the seasonal presence of fish. Bottom: seasonal patterns in water flow. Lines are fit by generalized additive models using the geom_smooth() function in the R package ggplot2.

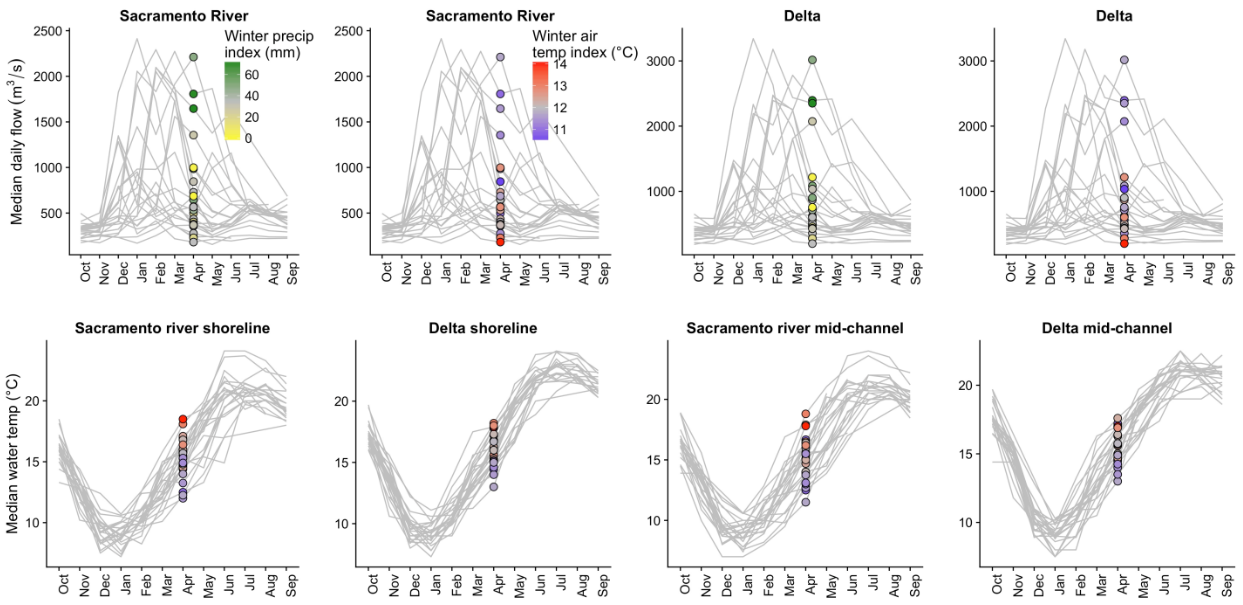

Figure S2. Flow and temperatures along shoreline and mid-channel waters of the Upper Sacramento River and the Delta. Points are placed on April and colored to indicate winter precipitation and air temperature conditions.

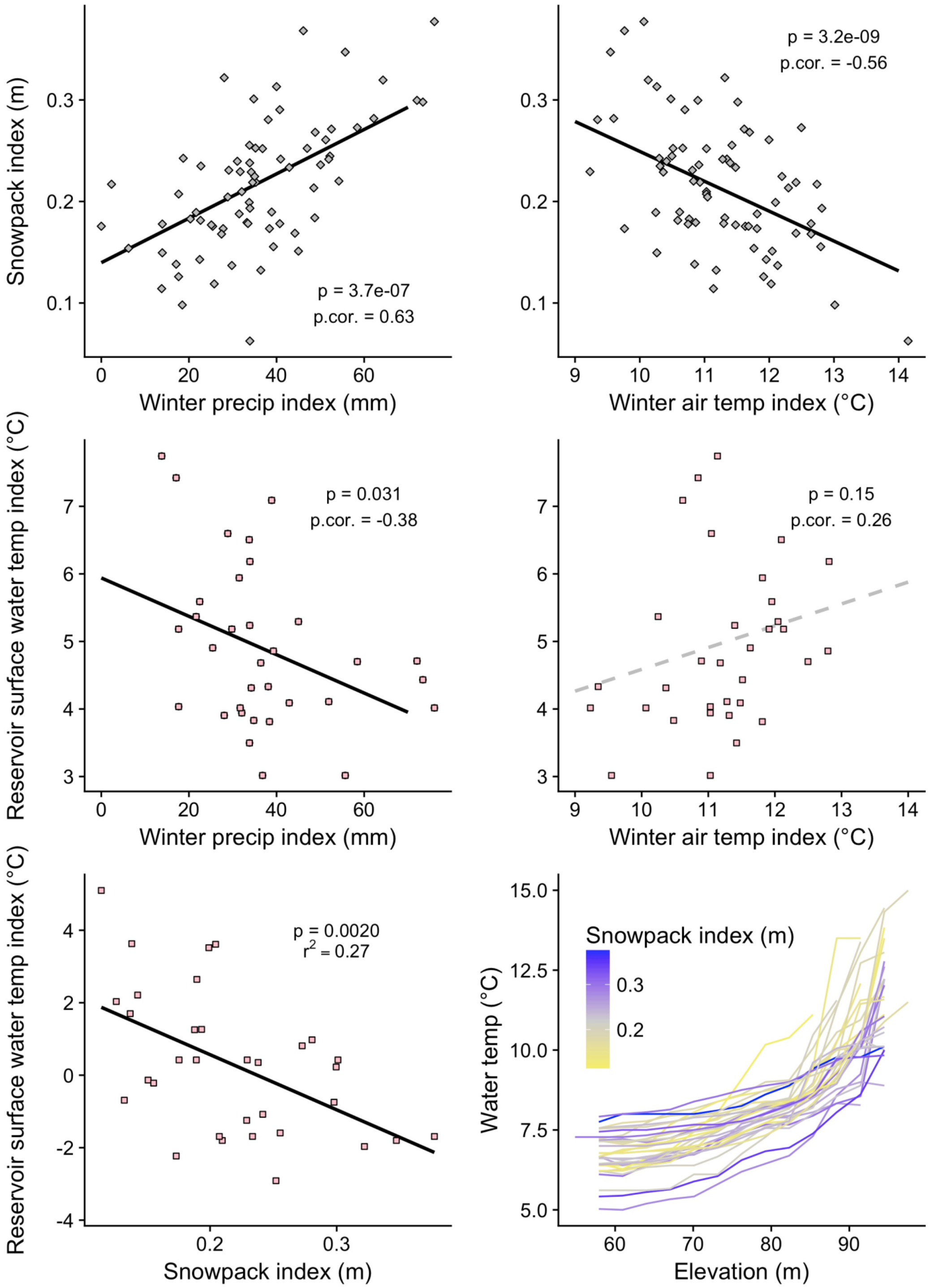

Figure S3. Springtime reservoir conditions compared to winter precipitation and air temperature. Lines indicate relationships predicted by linear models for variables shown on the x and y axes while holding the other explanatory variables at their median values. Lines are solid black for statistically significant ($\alpha < 0.05$) relationships and we report correlations, partial correlations, and p-values for relationships between the variables shown on the x and y axes.

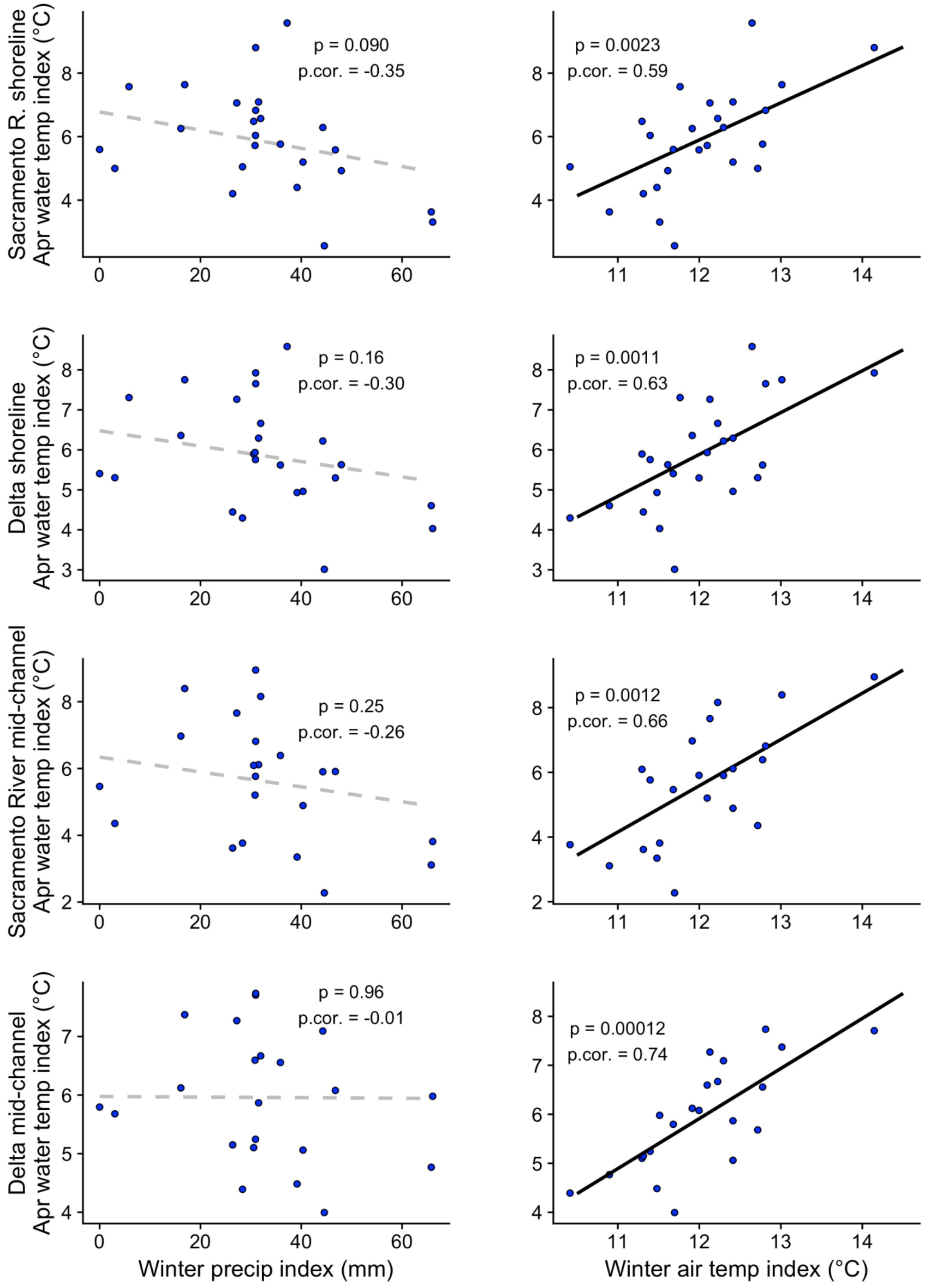

Figure S4. Springtime temperature conditions compared to winter precipitation and air temperature. Lines indicate relationships predicted by linear models for variables shown on the x and y axes while holding the other explanatory variables at their median values. Lines are solid black for statistically significant ($\alpha < 0.05$) relationships and we report correlations, partial correlations, and p-values for relationships between the variables shown on the x and y axes.

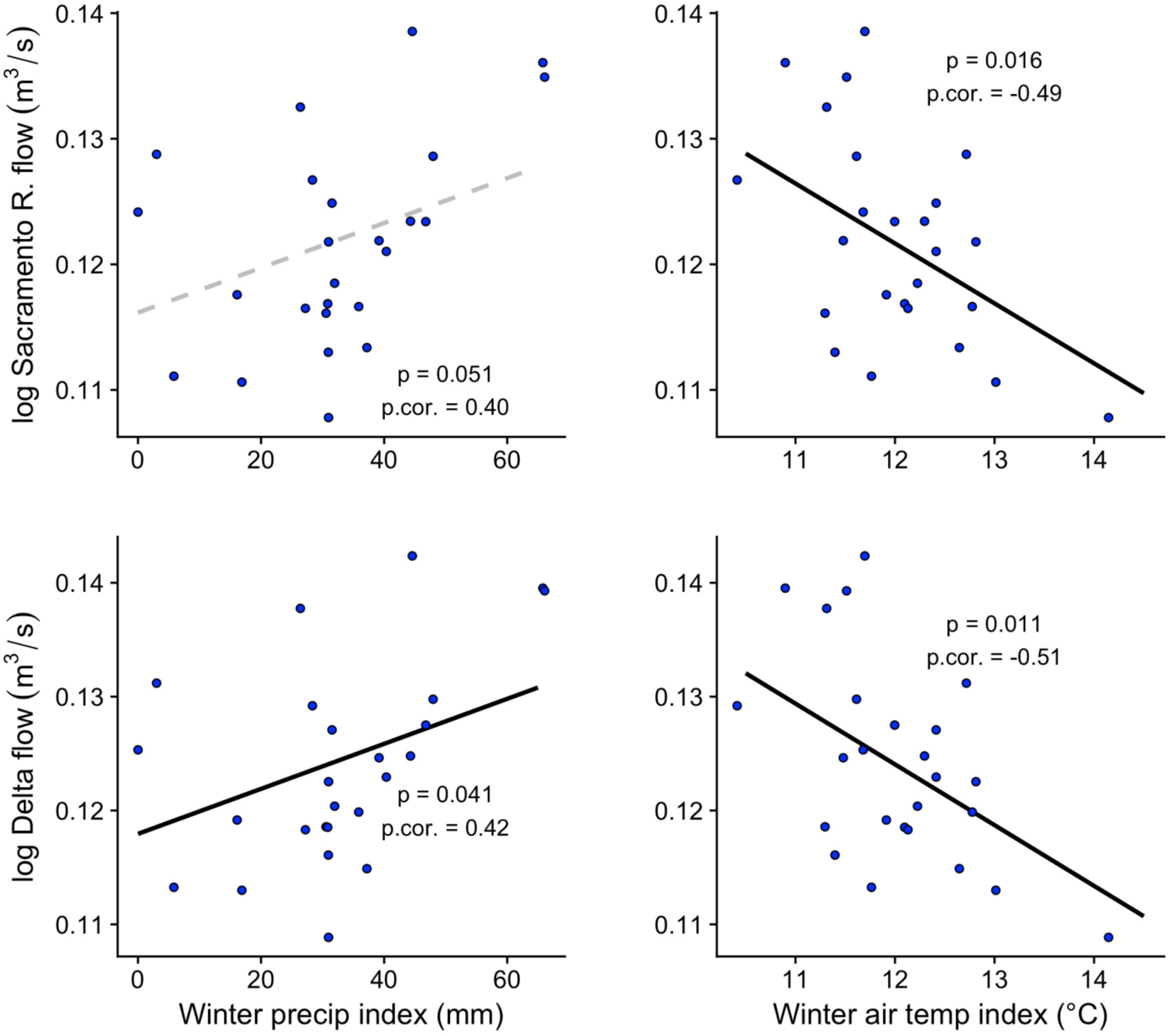

Figure S5. Springtime flow conditions compared to winter precipitation and air temperature. Lines indicate relationships predicted by linear models for variables shown on the x and y axes while holding the other explanatory variables at their median values. Lines are solid black for statistically significant ($\alpha < 0.05$) relationships and we report correlations, partial correlations, and p-values for relationships between the variables shown on the x and y axes.
